# Cyclocarya paliurus leaves extracts alleviate metabolic phenotypes in Chinese T2DM patients by modulating gut microbiota and metabolites: a clinical randomized controlled trial

**DOI:** 10.3389/fendo.2023.1176256

**Published:** 2023-05-24

**Authors:** Xiaojuan Peng, Sisi Chen, Lu Zhong, Yuting Li, Chutian Wu, Lixian Zhong, Weiwei Chen, Jinying Yang, Jiahua Zeng, Shaohui Tang

**Affiliations:** ^1^ Department of Endocrinology, Liuzhou People’s Hospital, Liuzhou, Guangxi, China; ^2^ Department of Gastroenterology, The First Affiliated Hospital, Jinan University, Guangzhou, Guangdong, China; ^3^ Department of Endocrinology, Affiliated Hospital of Xiangnan University, Chenzhou, Hunan, China

**Keywords:** Cyclocarya paliurus leaves extracts (CP), type 2 diabetes mellitus (T2DM), randomized controlled trial (RCT), metabolic phenotypes, gut microbiota

## Abstract

**Objective:**

We aimed to investigate the effect of Cyclocarya paliurus leaves extracts (CP) on glucose and blood lipid metabolism and its relationship with intestinal flora in type 2 diabetes mellitus (T2DM) patients.

**Methods:**

In this open-label, 84-day randomized controlled trial, a total of 38 T2DM patients were randomly assigned to the CP group or the Glipizide group (G group) in a 2:1 ratio. T2DM-associated metabolic phenotypes, gut microbiota and metabolites including short-chain fatty acids (SCFAs) and bile acids (BAs) were detected.

**Results:**

At the end of intervention, CP, like Glipizide, significantly improved HbA1c level and other glucose metabolism parameters (fasting plasma glucose (FBG), 2-hour post-meal blood glucose (2hPBG), the area under curve of oral glucose tolerance test glucose (OGTT glucose AUC)). Moreover, CP also resulted in the significant improvement in the levels of blood lipid and blood pressure. Notably, the improvement in blood lipid(triglycerides (TG) and high-density lipoprotein cholesterol (HDL-c)) and blood pressure (diastolic blood pressure (DBP)) was significantly greater in the CP group compared with the G group. Furthermore, the liver and kidney function parameters did not significantly change in both CP group and the G group over the 84-day period. Additionally, the enrichment of potentially beneficial bacteria (Faecalibacterium and Akkermansia), SCFAs and unconjugated BAs and the depletion of potential pathogenic bacteria (Prevotella_9) and conjugated BAs were observed in the CP group, while the abundances of the gut microbial were kept stable in the G group after intervention.

**Conclusion:**

CP displays a more beneficial effect in the alleviation of T2DM-associated metabolic phenotypes than glipizide by regulating gut microbiota and metabolites in T2DM patients, with no significant effects on liver and kidney function.

## Introduction

Type 2 diabetes mellitus (T2DM), whose prevalence has been estimated to raise from 536.6 million people in 2021 to 783.2 million by 2045 (1), causes lots of linked complications, such as kidney failure, heart disease, amputation, and blindness (2), and a heavy burden on public health. At present, the two main methods to control T2DM are lifestyle changes and drug therapy (3). Although there are multiple anti-diabetic medications, it is still difficult to control the progression of T2DM. Additionally, anti-diabetic medications have many unwanted side effects such as lactic acidosis and hypoglycemia, and ultimately fail to control blood glucose levels (4). Therefore, it is necessary to find out safer and more effective antidiabetic medications for T2DM.

Cyclocarya paliurus (C. paliurus) (Batal) IIjinskajia, an endemic plant belonging to Cyclocarya genus of the Juglandaceae family, is also known as the money tree, and it is distributed in 420–2500 m mountainous regions of Jiangxi, Hubei, Hunan, Guizhou and other provinces in China (5). Studies have shown that the leaves of C. paliurus contain a variety of bioactive compounds, such as polysaccharides, flavonoids, triterpenoids and phenolic compounds (6, 7). It has been reported that C. paliurus can reduce the levels of blood glucose, blood lipid and blood pressure in rats (8, 9). Based on its characteristics, in 1999, the United States Food and Drug Administration (FDA) approved the leaves of C. paliurus as a dietary supplement product and it is the first Chinese herbal tea approved by the institution (10). Therefore, C. paliurus has attracted more and more attention, and several studies found that polysaccharides and polyphenols (flavonoids, tannins, phenolic acids, and anthocyanins) isolated from C. paliurus leaves exert a potent hypoglycemic effect to ameliorate high-fat diet-low dose streptozotocin (STZ)-induced experimental T2DM in mice (11, 12). However, the mechanism of the effect of bioactive constituents of C. paliurus leaves on glucose and blood lipid metabolism improvement is still unclear.

Gut microbiota plays an important role in T2DM. Moderate dysbiosis with a decrease in butyric-producing bacteria and an increase in opportunistic pathogens was found in T2DM patients in China (13). Recently, accumulating evidence demonstrated that C. paliurus could impact T2DM through gut microbiota and its metabolites. Li et al. found that polysaccharide from C. paliurus leaves attenuated diabetes through multi-path of gut microbiota and host metabolism in T2DM rats (14). Yao et al. revealed that C. paliurus alleviate T2DM symptoms by modulating gut microbiota and short-chain fatty acids (SCFAs) in T2DM rats (15).

As above mentioned, C. paliurus seems to have the potential to be a new treatment medication for T2DM. However, to our knowledge, no existing literature or research is available to address the question concerning whether C. paliurus can improve glucose and lipid metabolism in T2DM patients. Thus, we conducted a pilot clinical randomized controlled study to compare the effect of C. paliurus leaves extracts (CP) and an antidiabetic drug Glipizide on metabolic phenotypes, including the intestinal microbiota and their metabolites (SCFAs and bile acids) in T2DM patients.

## Method

### Study design and subjects

A randomized and open-label, controlled clinical trial was designed and conducted according to Consolidated Standards of Reporting Trials guidelines (16). Participants were all recruited from the community healthcare center at West Renmin Road, Yanquan Road and Xiameiqiao Road in Chenzhou, Hunan, China from January 2019 to December 2020. All participants with T2DM signed written informed consentforms before enrollments. The trial protocol was registered at Chinese Clinical Trial Registry (http://www.chictr.org.cn/) and got a registration number of ChiCTR1900020482. Chinese newly onset, treatment-naive T2D patients from Han nationality with the age of 35- to 70-year old were enrolled in this trial. The inclusion criteria were as follows: (1) fasting plasma glucose (FBG) ≥7 mmol/L or*/*and 2-h oral glucose tolerance test ≥11.1 mmol/L; (2) HbA1c ≥6.5% (6.5%≦HbA1c≦12%) (17, 18). The exclusion criteria were listed in **Table S1** (**Supplementary Material Content 1**). The ethics committee of Affiliated Hospital of Xiangnan University approved the study protocol (Ethical approval department No. KY-20181226001).Patients who met all inclusion criteria were randomly assigned to either the C. paliurus leaves extracts group (CP group) or the Glipizide group (G group) in a 2: 1 ratio using a computer-generated random number allocation by a researcher not involved in the study.

### Sample sizes calculation

According to Movahed et al., the HbA1c difference between intervention group and control group was expected to be 1.2% at the end of the intervention (19). Combined with a standard deviation of 1.09%, α=0.05 and power=0.80, we calculated that a minimum of 11 T2DM patients were required in each group. Assuming a shedding rate of 10%, each group needed12 T2DM patients at least according to the actual situation. For this clinical trial, a minimum of 36 T2DMpatients were required.

### The medication intervention

Before starting the clinical trial, all the enrolled participants underwent a 2-week run-in period and received a diet and exercise education about the daily management of T2DM. Once the 2-week run-in period completed, participants entered the intervention study immediately. In the CP group, the participants received CP powder (2g/time, 3 times/day orally before meals) (CP were provided by Chenzhou Mingrun biological products Co. LTD, Hunan, China) (**Figure S1**). In the G group (the positive-control group), the participants received Glipizide Controlled-Release Tablets (5mg/time, 2 times/day orally before meals) (Pfizer Pharmaceuticals LLC, hereinafter referred to as Glipizide) that has no significant effect on gut microbiota of T2DM patients (20). During the intervention, all participants were required to monitor FBG, and 2-hour post-meal blood glucose (2hPBG) using Roche glucometer (Accu-Chek Performa) every day.

### Preparation of Cyclocarya paliurus leaves extracts and phytochemical analysis

Cyclocarya paliurus leaves extracts (CP)were provided by Chenzhou Ming run biological products Co. LTD Hunan, China. The license number of this product is QS431006011417.The leaves of C. paliurus has long been used as a bitter Traditional Chinese Medicine which has also been historically used as an herbal tea in the folk. Nowadays, the products derived from the leaves of C. paliurus have become a very popular health product in China (11). CP were prepared according to the method described previously with some modifications (21). Briefly, the leaves of Cyclocarya paliurus were dried and pulverized before extraction. Cyclocarya paliurus leaves powders were soaked with distilled water at 90 °C for 1 h (1:30, g/mL), and the extraction solution was centrifuged at 4500 × g for 15 min. The above operation was repeated once. The supernatants (extraction solutions, two times) were combined and concentrated using a rotary evaporator under vacuum. The resulting residue was dissolved with deionized water, filtered through a 800 mesh standard sieve. The elution was performed using 80% ethanol and the effluent of ethanol solution was collected and concentrated, resulting in the CP.

The composition of the C. paliurus leaves extracts was determined by high performance liquid chromatography (HPLC, Agilent 1200, USA) equipped with Agilent 5 TC-C18 (4.6 mm ×250 mm, 5μm). The system parameters were set as follows: injection volume, 5μL; the column temperature, 30°C; and the mobile phase flow rate, 0.8mL/min. The mobile phase consisted of two solvents: (A) 0.2% aqueous acetic acid and (B) acetonitrile. The elution conditions were as follows: 0–5min, gradient 5% B; 5–10 min, linear gradient 5–10% B; 10–15 min, linear gradient 10–25% B; 15–25 min, linear gradient 25–40% B; 25–30min, linear gradient 40–90% B. Peaks were detected at 254 nm. The quantification of different component monomers was based on the peak areas and calculated as equivalents of the standard compounds; all contents were expressed as mg/g component dry weight. The temperature of the column oven was set at 40 °C, the flow rate was set at 0.6 mL/ min and the injection volume was 5 µL.

### Clinical assessment

Participants were asked not to make any change to their daily physical activity and to maintain body weight. On Day 0, Day 42, and Day 84, the 7-day recall method was used to measure physical activity energy expenditure through an interview-administered survey instrument modified from the Cross-Cultural Activity Participation Study (22). Metabolic Equivalent Tasks (METs) were conducted to measure the physical activity energy expenditure of all the participants. On Day 0 and Day 84, the medical history and anthropometric evaluation (body weight (BW), waist circumference (WC), waist to hip ratio (WHR), body mass index (BMI), systolic blood pressure (SBP) and diastolic blood pressure (DBP)) were recorded and fasting blood samples and fecal samples were collected. Oral glucose tolerance test(OGTT), insulin release test and C-Peptide release test were also performed. Further details about the physical activity assessment can be found online (**Supplementary Methods, Supplementary Material Content 2**). Adverse effects were also recorded by the research team.

### Clinical outcomes

The primary clinical outcome was a change in the level of HbA1c over 84 days in the two treatment groups. The secondary clinical outcomes included the changes in the levels of FBG, 2hPBG, the area under curve of OGTT blood glucose, fasting insulin (FINS), 2-hour post-meal insulin (2hPINS), the area under curve of insulin release test, fasting C- peptide (FCP), 2-hour post-meal C-Peptide (2hPCP), the area under curve of C-peptide release test, total cholesterol (TC), triglycerides (TG), low-density lipoprotein cholesterol (LDL-c), high-density lipoprotein cholesterol (HDL-c), BW, WC, WHR, BMI, SBP, and DBP.

### Gut microbiome, short-chain fatty acids and bile acids analysis

The stool samples were immediately transferred to the -80°Cfreezer for storage after collection on day 0 and day 84. Then the stool samples were used for bacterial 16sRNA gene sequencing, and SCFAs as well as BAs analysis. The detailed information was shown on **Supplementary Material Content 2**.

### Safety assessment

The liver function (the total bilirubin (TBIL), direct bilirubin (DBIL), indirect bilirubin (IBIL), albumin (ALB), alanine aminotransferase (ALT), aspartate aminotransferase (AST)) and renal function (serum creatinine (Scr) and blood urea nitrogen (BUN))of all participants was assessed on day 0 and day 84.

### Fecal microbiota transplantation in pseudo-sterile mice

The animal experimental protocol was approved by the animal ethics committee of Jinan University (20190821-02). A total 50 of C57BL/6J mice (weight, 25.00 ± 2g; age, 10 weeks) were purchased from Biotechnology Company Limited (Beijing, China). The mice were raised in the specific pathogen-free animal experiment center at Jinan University, with constant temperature (24 ± 2°C), constant humidity of 55 ± 10% and a 12-h light/dark cycle. All mice received adaptive feeding for 7 days, and then were randomly divided into a control group (NC, n=10) and an antibiotic treatment group (AT, n=40). Within the next 4 weeks, the mice in AT group were fed with antibiotic-containing sterile water and then divided into 4 groups. More detailed information was shown in **Supplementary Material Content 2**.

### Statistical analysis

Outcomes were calculated on a strict intention-to-treat (ITT) basis. The raw data of Day 84 – the raw data of Day 0 represented the changes in all variables. The Kolmogorov-Smirnov Test was used to detect whether the continuous data conform to normal distribution. If normally distributed, paired T-test was used for intra-group comparison and independent T-test was used for inter-group comparison, and the data were expressed as mean and standard deviations. If not normally distributed, non-parametric two-tailed Wilcoxon matched-pairs signed-ranks was used for intra-group comparison and non-parametric K-independent Wilcoxon signed-ranks were used for inter-group comparison, and the data were expressed as the median and inter-quartile ranges. Fold changes of diabetes-induced markers after treatments of CP & G were calculated as (the raw data of day 84/the raw data of day 0). Statistical Package for Social Science (SPSS) 28.0 was used to analyze all data and we defined *P*<0.05 as statistically significant. All data visualization were made by GraphPad Prism (version 9.0). We used the OmicStudio tools at https://www.omicstudio.cn.to make a clustering correlation heatmap.

## Results

### Phytochemical characterization of CP

Phytochemical compositions of the CP were clarified by using high performance liquid chromatography (HPLC). The 5 components in the CP were identified in the chromatogram with peaks at 17.19 min to3-cafeoylquinic acid, 20.19 min to isoquercitrin, 22.19 min to kaempferol-3-glucoside, 23.43 min to kaempferol 3-rhamnoside, 27.56 min to quercetin by the comparison to external standards (**Figure S2**). Also, the contents of five compounds in the CP were measured by HPLC and their contents were 53.4, 42.5, 92.6, 4.8 and 2.1 mg/g in the CP, successively (**Table S2, Supplementary Material Content 1**).

### Clinical baseline characteristics and physical activity of participants

According to the inclusion and exclusion criteria, a total of 38 participants with T2DM were randomly divided into the G group (n=13) and CP group (n=25) in the study. During the intervention, 6 participants dropped out (1 participant in the G group and 5 participants in the CP group). Finally, a total of 32 participants completed the intervention (**Figure S3**). At baseline, anthropometric parameters (BW, WC, WHR, BMI, SBP and DBP), blood glucose homeostasis (HbA1c, FBG, 2hPBG, FINS, 2hPINS, HOMA-IR, Fasting C-Peptide, 2hPCP, OGTT glucose AUC, Insulin release test AUC, C-Peptide release test AUC), blood lipid homeostasis (TG, TC, LDL-c and HDL-c) and other blood markers (TBIL, DBIL, IBIL, ALT, AST, ALB, Scr, BUN) were not statistically different between the two groups (**Table S3, Supplementary Material Content 1**). In addition, there were no significant differences in physical activity levels in the intra-group and inter-group comparisons on Day 0 and on Day 84 (**Table S4, Supplementary Material Content 1**).

### CP alleviates HbA1c level in T2DM patients

**Figure 1:**
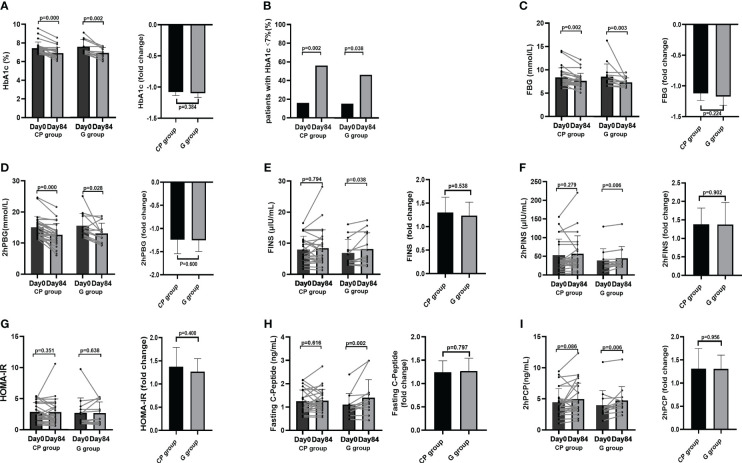
The changes in glucose metabolism phenotypes in T2DM patients. **(A)** The change in HbA1c level. **(B)** The percentage of participants with HbA1c<7%. **(C-I)** The changes in the levels of FBG **(C)**, 2hPBG **(D)**, FINS **(E)**, 2hPINS **(F)**, HOMA-IR **(G)**, Fasting C-Peptide **(H)**, 2hPCP **(I)**. Data are presented as means ± SD or medians (IQRs). Paired T-test or non-parametric two-tailed Wilcoxon paired sign rank test was used for intra-group comparisons and independent T-test or non-parametric K independent Wilcoxon signed-ranks was used for comparisons of the fold changes between the two groups. CP group, cyclocarya paliurus leaves extracts group; G group, Glipizide group; SD, standard deviation; IQR, interquartile range; T2DM, type 2 diabetes mellitus; FBG, fasting blood glucose; FINS, fasting insulin; 2hPBG, 2-hour post-meal blood glucose; 2hPINS, 2-hour post-mealinsulin;HOMA-IR, homeostasis model assessment for insulin resistance = (fasting blood glucose×fasting insulin/22.5), 2hPCP, 2-hour post-meal C-Peptide.

Compared with Day 0, the level of HbA1c, the primary clinical outcome, was significantly decreased both in the G group and in the CP group on Day 84. At the end of intervention, there was no significant difference in the reduction of HbA1c between the two groups (**Figure 1A**; **Table 1**). We also calculated the proportion of participants who achieved adequate glycemic control (HbA1c<7%). On Day 0, there were no significant differences in the proportion of participants who achieved HbA1c<7% between the two groups, and both the G group and the CP group had a significant increase in the proportion of participants who achieved HbA1c<7% on Day 84 compared with Day 0. We also found that there was no significant difference in the proportion of participants who achieved HbA1c<7% on Day 84 between the two groups (**Figure 1B**; **Table S5, Supplementary Material Content 1**). These results demonstrate that the CP induce a significant reduction in HbA1c, which is similar to the effect of Glipizide on the reduction of HbA1c in T2DM patients.

**Table 1:** Comparison of metabolic parameters in T2DM patients.

Outcomes	CP group			G group			*p*
	Day 0 (n=25)	Day 84 (n=25)	*P-value*	Day 0 (n=13)	Day 84 (n=13)	*P-value*	
Blood glucose homeostasis							
HbA1c (%)	7.29 (7.07, 7.63)	6.70 (6.50, 7.28)	0.000	7.14 (7.08, 8.44)	7.01 (6.42, 7.55)	0.002	0.384
FBG (mmol/L)	8.37±2.05	7.64±1.62	0.002	7.80±1.26	6.73±0.98	0.003	0.224
2hPBG (mmol/L)	14.56 (12.47, 16.58)	12.72 (10.27, 14.05)	0.000	14.45 (12.60, 19.00)	12.8 (10.05, 16.08)	0.028	0.600
OGTT glucose AUC (mmol/L/min)	2226.15 (1972.72, 2688.37)	1925.55 (1733.40, 2352.15)	0.000	2258.10 (2026.90, 2827.05)	2063.70 (1838.62, 2317.87)	0.008	0.877
FINS (μIU/mL)	6.35(4.39,10.93)	7.42(4.02,11.22)	0.794	4.46 (3.40, 9.80)	5.82 (3.51, 13.42)	0.038	0.538
2hPINS (μIU/mL)	37.34 (27.02, 85.43)	40.48 (27.33, 75.23)	0.279	26.77 (21.56, 51.74)	32.34 (28.07, 55.25)	0.006	0.902
Insulin release test AUC (μIU/mL/min)	4942.2 (3613.35, 10822.20)	5579.55 (3932.55, 11280.75)	0.145	4249.2 (2807.7, 5489.25)	4915.5 (3871.05, 5895.75)	0.004	0.195
HOMA-IR	2.26 (1.65, 4.45)	2.11 (1.38, 3.97)	0.351	1.46 (1.24, 3.18)	1.96 (1.01, 3.77)	0.638	0.400
Fasting C-Peptide (ng/mL)	1.11 (0.88, 1.73)	1.22 (0.94, 1.56)	0.616	0.99 (0.85, 1.30)	1.21 (0.93, 1.57)	0.002	0.797
2hPCP (ng/mL)	3.82 (3.15, 5.11)	4.47 (3.17, 6.51)	0.086	3.53 (2.83, 4.40)	4.58 (3.67, 4.99)	0.006	0.956
C-Peptide release test AUC (ng/mL/min)	541.80 (419.60, 711.80)	576.6 (462.40, 891.00)	0.064	420.750 (378.45, 634.20)	579.150 (516.00, 640.80)	0.015	0.453
Blood lipid homeostasis							
TG(mmol/L)	2.38±1.51	1.98±1.01	0.014	1.67±0.92	1.80±0.77	0.250	0.014
TC(mmol/L)	5.65±1.35	5.13±1.26	0.001	4.54±089	4.28±0.90	0.064	0.672
LDL-c(mmol/L)	3.40±1.24	2.88±1.15	0.007	2.55±0.71	2.44±0.85	0.231	0.735
HDL-c(mmol/L)	1.17±0.42	1.27±0.33	0.006	1.22±0.32	1.16±0.33	0.523	0.043
Anthropometric markers							
BW (kg)	60.88±7.95	60.66±8.06	0.408	63.1±8.36	62.69±8.19	0.203	0.955
WC (cm)	87.16±5.74	87.03±6.14	0.817	89.88±6.80	88.92±7.14	0.126	0.589
WHR	0.92±0.38	0.93±0.47	0.298	0.94±0.50	0.93±0.51	0.231	0.421
BMI	23.95±2.07	23.82±2.01	0.165	24.38±2.33	24.23±2.34	0.229	0.853
SBP (mmHg)	136.08±19.23	129.80±16.25	0.011	125.84±19.13	121.30±16.73	0.116	0.820
DBP (mmHg)	81.00 (73.00, 88.50)	74.00 (68.50, 82.50)	0.001	67.00 (60.00, 87.50)	73.00 (63.00, 77.00)	0.789	0.028
Liver and renal function							
TBIL (umol/L)	16.34±5.38	17.74±496	0.149	16.82±5.00	15.62±5.02	0.334	0.511
DBIL (umol/L)	4.80 (3.35, 6.05)	4.60(3.45, 5.60)	0.681	5.20 (4.95, 6.45)	5.00 (4.10, 6.90)	0.386	0.557
IBIL (umol/L)	11.00(9.15, 13.00)	9.80 (8.25,11.25)	0.097	9.90 (9.20, 12.35)	10.30 (7.60, 11.20)	0.666	0.566
ALT (U/L)	19.00 (15.00, 27.00)	18.00 (13.50,23.50)	0.092	23.00 (15.00, 25.50)	21.00 (14.50, 28.00)	0.397	0.164
AST (U/L)	19.00 (16.50, 22.50)	20.00 (17.00, 22.50)	0.749	19.00 (17.50, 25.50)	18.00 (17.00, 22.50)	0.180	0.331
ALB (g/L)	43.73±1.91	43.76±2.28	0.347	43.53±2.88	44.54±3.74	0.295	0.123
Scr(umol/L)	70.47±15.51	70.06±14.62	0.721	69.9±13.44	66.96±10.27	0.286	0.136
BUN(mmol/L)	5.15±1.10	5.39±1.14	0.199	4.66±1.03	4.87±1.19	0.465	0.702

The data are shown as the mean ± SD for normal variables or median (IQR) for non-normal variables. Paired T-test or non-parametric two-tailed Wilcoxon paired sign rank test was used for intra-group comparisons. P-value, comparison between Day 0 and Day 84 at the same group. Independent T-test and non-parametric K independent Wilcoxon signed-ranks was used for comparisons of the changes between the two groups. P, comparison of the fold changes between the CP group and the G group.

CP group, cyclocarya paliurus leaves extracts group; G group, Glipizide group; SD, standard deviation; IQR, interquartile range; HbA1c, hemoglobin A1c; FBG, fasting blood glucose; 2hPBG, 2-hour post-meal blood glucose; FINS, fasting insulin; 2hPINS, 2-hour post-meal insulin; HOMA-IR, homeostasis model assessment for insulin resistance = (fasting blood glucose×fasting insulin/ 22.5); 2hPCP, 2-hour post-meal C-Peptide; OGTT, oral glucose tolerance test; AUC, area under curve; TG, triglycerides; TC, total cholesterol; LDL-c, low-density lipoprotin cholesterol; HDL-c, high-density lipoprotein cholesterol; BW, body weight; WC, waist circumference; WHR, waist to hip ratio; BMI, body mass index; SBP, systolic blood pressure; DBP, diastolic blood pressure; TBIL, total bilirubin; DBIL, direct bilirubin; IBIL, indirect bilirubin; ALB, albumin;ALT, alanine aminotransferase; AST, aspartate aminotransferase; Scr, serum creatinine; BUN, blood urea nitrogen.

### CP ameliorates several other metabolic phenotypes in T2DM patients

**Figure 2:**
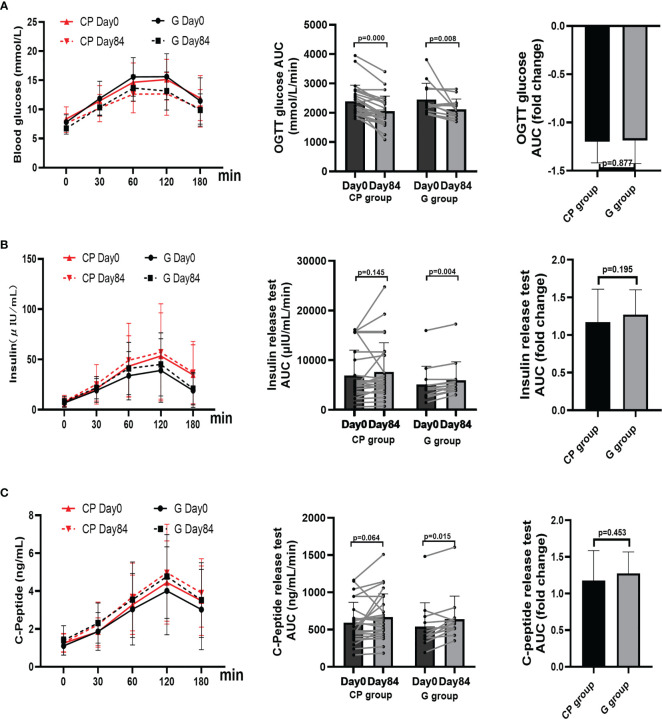
The changes in OGTT glucose AUC, insulin release test, and C-Peptide release test in T2DM patients. **(A–C)** The changes in the levels of OGTT glucose AUC **(A)**, insulin release test AUC **(B)**, and C-Peptide release test AUC **(C)**. Data are presented as means ± SD or medians (IQRs). Paired T-test or non-parametric two-tailed Wilcoxon paired sign rank test was used for intra-group comparisons and independent T-test or non-parametric K independent Wilcoxon signed-ranks was used for comparisons of the fold changes between the two groups. CP group, cyclocarya paliurus leaves extracts group; G group, Glipizide group; SD, standard deviation; IQR, interquartile range; AUC, area under curve.

**Figure 3:**
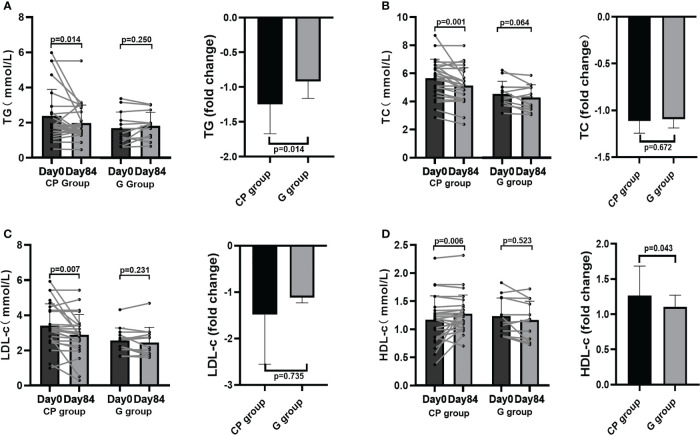
Blood lipid homeostasis in T2DM patients. **(A–D)** The changes in the levels of TG **(A)**, TC **(B)**, LDL-c **(C)**, HDL-c **(D)**. Data are presented as means ± SD or medians (IQRs). Paired T-test or non-parametric two-tailed Wilcoxon paired sign rank test was used for intra-group comparisons and independent T-test or non-parametric K independent Wilcoxon signed-ranks was used for comparisons of the fold changes between the two groups. CP group, cyclocarya paliurus leaves extracts group; G group, Glipizide group; SD, standard deviation; IQR, interquartile range; TG, triglycerides; TC, total cholesterol; LDL-c, low-density lipoprotin cholesterol; HDL-c, high-density lipoprotein cholesterol.

From Day 0 to Day 84, the significant improvement in blood glucose homeostasis (FBG, 2hPBG, OGTT glucose AUC) (**Table 1**; **Figures 1C, D**, **2A**), blood lipid homeostasis (TG, TC, LDL-c and HDL-c) (**Table 1**; **Figures 3A–D**) and anthropometric parameters (SBP and DBP) (**Table 1**; **Figures S4E, F**)was observed, whereas the other blood glucose homeostasis (FINS, 2hPINS, HOMA-IR, fasting C-peptide, 2hPCP, insulin release test AUC and C-peptide release test AUC) (**Table 1**; **Figures 1E–I**, **2B, C**) and anthropometric parameters (BW, WC, WHR and BMI) (**Table 1**; **Figures S4A–D**) were not significantly affected in the CP group. From Day 0 to Day 84, the significant amelioration in blood glucose homeostasis (FBG, 2hPBG, OGTT glucose AUC, FINS, 2hPINS, fasting C-peptide, 2hPCP, insulin release test AUC and C-peptide release test AUC) was found, while the other blood glucose homeostasis (HOMA-IR), blood lipid homeostasis (TG, TC, LDL-c and HDL-c) and anthropometric parameters (BW, WC, WHR and BMI) were not significantly affected in the G group. The fold changes in blood lipid homeostasis (TG and HDL-c) and anthropometric parameters (DBP) was significantly greater in the CP group compared with the G group. Moreover, there were no significant differences in the fold changes in the other blood glucose homeostasis (FBG, 2hPBG, FINS, 2hPINS, fasting C-peptide, 2hPCP, HOMA-IR, OGTT glucose AUC, insulin release test AUC and C-peptide release test AUC), blood lipid homeostasis (TC and LDL-c) and anthropometric parameters (BW, WC, WHR, BMI and SBP) over the 84-days between the two groups. Also, the liver and kidney function parameters (TBIL, DBIL, IBIL, ALT, AST, ALB, Scr and BUN)did not significantly change in both CP group and the G group over the 84-day period (**Table 1**; **Figures 1**-**3**, **S4**, **5**).These results suggest that CP can alleviate T2DM-associated clinical markers, blood lipid and blood pressure in addition to alleviating HbA1c in T2DM patients, having no significant impacts on the liver and kidney function.

### CP redresses gut microbiota dysbiosis in T2DM patients

**Figure 4:**
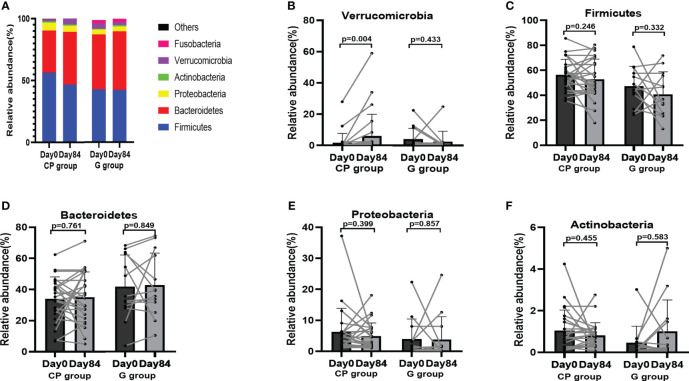
The abundances of the gut microbiota at the phylum level in T2DM patients. **(A)** The abundances of the gut microbiota at the phylum level. **(B-F)** The abundance of Verrucomicrobia **(B)**, Firmicutes **(C)**, Bacteroidetes **(D)**, Proteobacteria **(E)** and Actinobacteria **(F)**. Data are presented as means ± SD or medians (IQRs). Paired T-test or non-parametric two-tailed Wilcoxon paired sign rank test was used for intra-group comparisons. CP group, cyclocarya paliurus leaves extracts group; G group, Glipizide group; SD, standard deviation; IQR, interquartile range.

To explore the potential role of the gut microbiota in CP-induced metabolic phenotypes improvement in T2DM patients, 16S rRNA gene sequencing was performed in the fecal samples from the patients on Day 0 and Day 84. At the phylum level, the overall microbial compositions were shown in **Table S6** (**Supplementary Material Content 1**). The dominant species (mean relative abundance >1%) were Firmicutes, Bacteroidetes, Proteobacteria, Actinobacteria, Verrucomicrobia and Fusobacteria (**Figure 4A**). From Day 0 to Day 84, only the abundance of Verrucomicrobia was significantly increased, whereas there were no significant differences in the abundances of Firmicutes, Bacteroidetes, Proteobacteria, Actinobacteria, Fusobacteria, Synergistetes, Cyanobacteria and Patescibacteria in CP group. In the G group, the abundances of the gut microbial at the phylum level were kept stable during the intervention (**Table S6, Supplementary Material Content 1**; **Figures 4B–F**).

**Figure 5:**
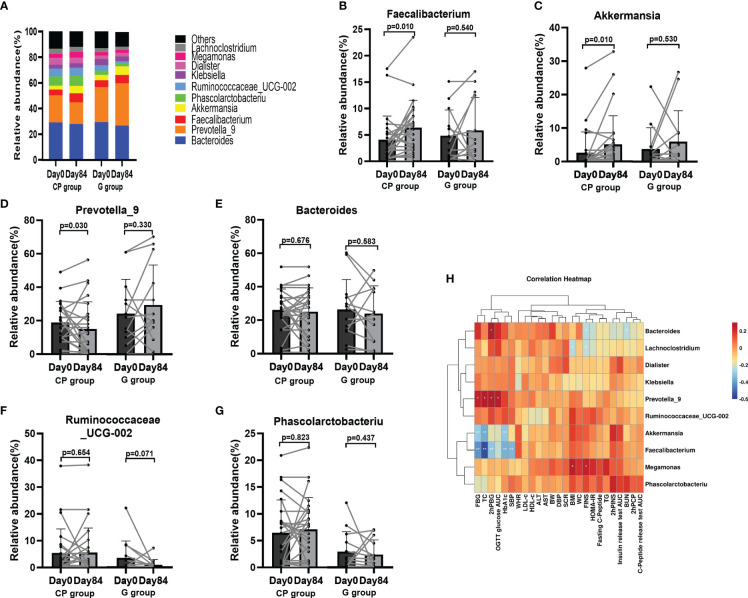
The abundances of the gut microbiota at the genus level in T2DM patients. **(A)** The abundances of the gut microbiota at genus level. **(B–G)** The abundance of Faecalibacterium **(B)**, Akkermansia **(C)**, Prevotella_9 **(D)**, Bacteroides **(E)** and Ruminococcaceae_UCG-002 **(F)** and Phascolarctobacteriu **(G)**. Data are presented as means ± SD or medians (IQRs). Paired T-test or non-parametric two-tailed Wilcoxon paired sign rank test was used for intra-group comparisons. **(H)** Theassociations between bacterial abundance at the genus level and metabolic parameters. CP group, cyclocarya paliurus leaves extracts group; G group, Glipizide group; SD, standard deviation; IQR, interquartile range; BW, body weight; WC, waist circumference; WHR, waist to hip ratio; BMI, body mass index; SBP, systolic blood pressure; DBP, diastolic blood pressure; HbA1c, hemoglobin A1c; FBG, fasting blood glucose; PBG, 2-hour post-meal blood glucose; FINS, fasting insulin; hPINS, 2-hour post-mealinsulin; 2hPCP, 2-hour post-mealC-Peptide; OGTT, oral glucose tolerance test; AUC, area under curve; HOMA-IR, homeostasis model assessment for insulin resistance = (fasting blood glucose×fasting insulin/22.5); TG, triglycerides; TC, total cholesterol; LDL-c, low-density lipoprotin cholesterol; HDL-c, high-density lipoprotein cholesterol, ALB, albumin; ALT, Alanine aminotransferase, Scr, serum creatinine; BUN, blood urea nitrogen.

At the genus level, the top 10 dominant genera was presented in **Figure 5A**. Compared with Day 0, the abundances of potentially beneficial bacteria (Faecalibacterium and Akkermansia) were significantly enriched, while the abundance of potentially pathogenic bacteria (Prevotella_9) was significantly reduced on Day 84 in the CP group (**Table S7, Supplementary Material Content 1**; **Figures 5B–D**). In addition, the abundances of other genera of bacteria showed no significant change in the CP group during the intervention. In the G group, the abundances of the gut microbiota at the genus level were not significantly affected from Day 0 to Day 84 (**Table S7, Supplementary Material Content 1**; **Figures 5E–G**).

Then we conducted a spearman correlation analysis to explore the potential relationships between bacterial abundance and metabolic phenotypes. We found that Faecalibacterium, which was enriched in the participants on Day 84 in the CP group, had a negative correlation with several metabolic phenotypes (HbA1c, FBG, 2hPBG, SBP and TC). Akkermansia, which was also enriched in the participants on Day 84 in the CP group, was negatively related to a few metabolic markers (HbA1c, FBG and TC). Prevotella_9 had a positive relationship with several metabolic markers (FBG, OGTT Glucose AUC, 2hPBG and TC) (**Figure 5H**). These findings indicate that CP-induced metabolic phenotype alleviation maybe associated with improved intestinal flora.

### CP induces metabolites alteration in T2DM patients

**Figure 6:**
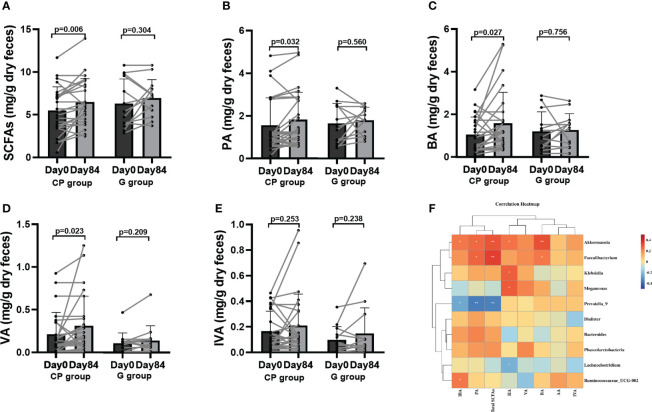
The levels of SCFAs in T2DM patients. **(A–E)** Total SCFAs **(A)**, PA **(B)**, BA **(C)**, VA **(D)** and IVA **(E)**. Data are presented as means ± SD or medians (IQRs). Paired T-test or non-parametric two-tailed Wilcoxon paired sign rank test was used for intra-group comparisons. **(F)** the associations between bacterial abundance and SCFAs. CP group, cyclocarya paliurus leaves extracts group; G group, Glipizide group; SD, standard deviation; IQR, interquartile range; SCFAs, short chain fatty acids; AA, acetic acid; PA, propionic acid; BA, butyric acid; IBA, isobutyric acid; VA, valeric acid; IVA, isovalerlc acid; HA, hexanoic acid.

To reveal metabolites alteration, related to the gut microbiota, which is potentially involved in CP-induced T2DM improvement, we performed metabolic profiling of feces from the participants. From Day 0 to Day 84, a significant increase was observed in total SCFAs, propionic acid (PA) and butyric acid (BA), while the levels of acetic acid (AA), isobutyric acid (IBA), valeric acid (VA), isovaleric acid (IVA) and hexanoic acid (HA) had no significant change in the CP group. In the G group, the level of total SCFAs, AA, PA, BA, IBA, VA, IVA and HA were not affected over the 84-day period (**Table S8, Supplementary Material Content 1**; **Figures 6A–E**).

**Figure 7:**
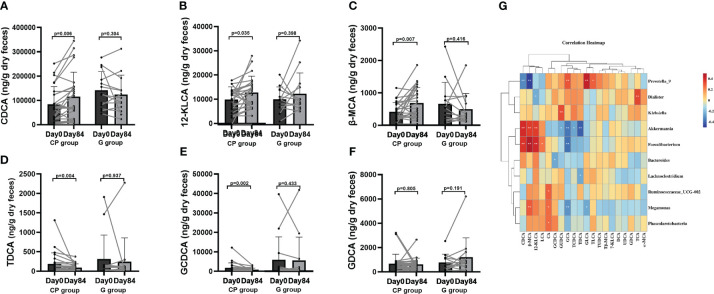
The levels of BAs in T2DM patients. **(A–F)** The changes in the levels of CDCA **(A)**,12-KLCA **(B)**, β-MCA **(C)**, TDCA **(D)**, GCDCA **(E)**, GDCA **(F)**. Data are presented as means ± SD or medians (IQRs). Paired T-test or non-parametric two-tailed Wilcoxon paired sign rank test was used for intra-group comparisons. **(G)** The associations between bacterial abundance and BAs. CP group, cyclocarya paliurus leaves extracts group; G group, Glipizide group; SD, standard deviation; IQR, interquartile range; BA, bile acids; CDCA, chenodeoxycholic acid; DCA, deoxycholic acid; LCA, lithocholic acid; UDCA, ursodeoxycholic acid; 7-KLCA, 7-ketolithocholic acid; CA, cholic acid; 12-KLCA, 12-ketolithocholic acid; β-MCA, β-muricholic acid; ω-MCA, ω-muricholic acid; GCDCA, glycochenodeoxycholic acid; GCA, glycocholic acid; TCA, taurocholic acid; TCDCA, taurochenodeoxycholic acid; GUDCA, glycoursodeoxycholic acid; TDHCA, taurodehydrocholic acid; GDCA, glycodeoxycholic acid; TUDCA, tauroursodeoxycholic acid; TLCA, taurolithocholic acid; Tβ-MCA, tauro-β-muricholic acid; GLCA, glycolithocholic acid; TDCA, taurodeoxycholic acid.

To explore the potential effect of CP on BA metabolism, a total of 20 species of BAs were detected. Compared to Day 0, the levels of unconjugated BAs (chenodeoxycholic acid (CDCA), 12-ketolithocholic acid (12-KLCA) and β-muricholic acid (β-MCA)) were significantly increased and the levels of conjugated BAs (taurodeoxycholic acid (TDCA),glycochenodeoxycholic acid (GCDCA) were significantly reduced, while no significant differences were found in other BAs in the CP group on Day 84. From Day 0 to Day 84, the levels of BAs showed no significant changes in the G group (**Table S9, Supplementary Material Content 1**; **Figures 7A–F**).

Correlation analyses were performed to determine the potential relationships between metabolites and gut microbiota. Coincidently, we observed that Faecalibacterium enriched in the CP group on Day 84 was positively related to SCFAs (total SCFAs, BA and PA) and unconjugated BAs (CDCA, 12-KLCA, β-MCA and LCA), but negatively correlated with conjugated BAs (GCA). Akkermansia, which was also enriched in the CP group on Day 84, showed a positive relationship with SCFAs (total SCFAs, BA, HA, IBA and PA), and unconjugated BAs (CDCA, 12-KLCA and β-MCA), but had a negative correlation with conjugated BAs (GUCDA, GCA, TDCA and TCDCA). Prevotella_9, which was depleted in the CP group on Day 84, was negatively correlated with SCFAs (total SCFAs, IBA and PA) and unconjugated BAs (CDCA and β-MCA), but positively associated with conjugated BAs (GCA, GLCA and TLCA). Megamonas was negatively correlated with conjugated BAs (GCA and GLCA), but positively associated with SCFAs (HA) and unconjugated BAs (β-MCA and CA) (**Figures 6F**, **7G**).Taken together, the abovementioned results reveal that the changes in gut microbiome abundances induced by CP may be responsible for the alteration of metabolites, thus affecting metabolic phenotypes in patients with T2DM.

### FMT ameliorates the glucose metabolism in pseudo-sterile mice

**Figure 8:**
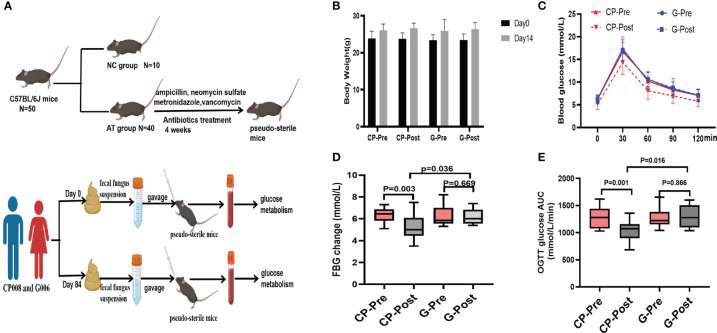
FMT improves glucose metabolism in pseudo-sterile mice. **(A)** Experimental design for animal study for microbiota transplantation. **(B)** Body weight. **(C)** OGTT blood glucose (2 weeks after transplantation) of mice receiving transplanted pre- and post-intervention human gut microbiota. **(D)** FBG level. **(E)** OGTT AUC. Paired T-test or non-parametric two-tailed Wilcoxon paired sign rank test was used for intra-group comparisons and independent T-test or non-parametric K independent Wilcoxon signed-ranks was used for comparisons of the changes between the two groups. CP group, cyclocarya paliurus leaves extracts group; G group, Glipizide group; SD, standard deviation; IQR, interquartile range; AT group, antibiotic treatment group; NC group, normal control group.

Experimental design for animal study for microbiota transplantation (**Figure 8A**). After the FMT, we observed that body weight was not also statistically significant both between intragroup and between intergroup (**Figure 8B**). Mice transplanted with the postintervention microbiota from the CP group showed better glucose metabolic parameters than those with the preintervention microbiota from the CP group, while mice transplanted with the postintervention microbiota from the G group showed no significant change in glucose metabolism parameters compared with the preintervention microbiota from the G group (**Figures 8C–E**).

## Discussion

In this pilot clinical randomized trial, we demonstrated for the first time that CP, like glipizide, significantly improved HbA1c level and other T2DM-associated glucose metabolism parameters, independent of physical activity and body weight and with no significant effects on liver and kidney function in the patients with T2DM. Moreover, CP also resulted in the improvement in the levels of blood lipid and blood pressure. These results indicate that CP displays a more beneficial effect in the alleviation of T2DM-associated metabolic phenotypes than Glipizide.

The improvements in T2DM-associated metabolic phenotypes induced by CP in the study may be explained in part by multiple chemical components in the CP, such as3-caffeoylquinic acid, isoquercitrin and kaempferol-3-glucoside. It has been shown that 3-caffeoylquinic acid is associated with a wide range of biological activities, such as antioxidation, antibacterial, antiparasitic, neuroprotective, anti-inflammatory, anticancer, antiviral, and antidiabetic effect (23). In the diabetic mice, isoquercitrin could significantly improve the sensitivity of adipose tissue to insulin and attenuate insulin resistance (24). Quercetin-3-glucuronide and kaempferol-3-glucuronideisolated from C. paliurus leaves showed potential anti-diabetic activity in STZ-stimulated mice (25). The above results were coincident with our findings that CP resulted in the alleviation of HbA1c and other T2DM-associated clinical markers and blood pressure, which was mainly associated with phytochemical compositions 3-caffeoylquinic acid, isoquercitrin and kaempferol-3-glucoside in the CP.

There is mounting evidence that gut microbiota involves the etiology and progression of T2DM, which has attracted the attention of researchers (26–28). Hence, we investigated the potential involvement in gut microbiota in mediating CP-induced T2DM improvement. In a multicenter, randomized, open label clinical trial, Chinese herbal formula alleviatedHbA1c, FBG, 2-h PBG, HOMA-IR, and TC levels through enriching Faecalibacteriumin T2DM patients with hyperlipidemia (29). Another study showed that participants with diabetes taking metformin had higher relative abundance of Akkermansia muciniphila and several SCFA-producing microbiota compared with participants without diabetes (30). In addition, a randomized, double-blind clinical trial showed that a freshwater fish-based diet ameliorated hepatic steatosis and other metabolic phenotypes by increasing Faecalibacterium abundance in patients with NAFLD (31). In agreement with human studies, the increase in the Akkermansia by metformin treatment contributed to the antidiabetic effects of metformin in diet-induced obese mice (32). Several clinical studies have found that Prevotell_9was enriched in patients with advanced liver fibrosis, liver cirrhosis, insulin resistance, type 2 diabetes, inflammation and obesity (33–35). In our study, we observed that the abundances of Faecalibacterium and Akkermansia were enriched, and the abundance of Prevotella_9 was decreased at the end of the intervention in the CP group, along with the improvement in HbA1c and other metabolic phenotypes. Supporting the aforementioned findings, our study provided new insights into the potential role of gut microbiota in mediating the more beneficial effect of CP on T2DM than Glipizide.

On the other hand, our results showed that Glipizide did not significantly affect the composition of intestinal flora during the intervention, which was consistent with the report by Wamg et al (20). Glipizide works by binding to the sulphonylurea receptor (SUR)-1 subunit of pancreatic β-cell ATP-sensitive potassium channels, leading to their closure; the resultant membrane depolarization opens voltage-dependent calcium channels, causing intracellular calcium concentrations to increase, with subsequent release of insulin (36).

In addition to gut microbiota, metabolites including SCFAs, amino acid catabolites, and BAs produced by gut microbiota play an important role in T2DM development. These metabolites alleviate or exacerbate T2DM via their homologous receptors-mediated signaling (27, 37). We therefore attempted to clarify the potential role of metabolites (SCFAs and BAs) in mediate the beneficial impact of CP on T2DM patients. The favorable effects of SCFAs on metabolic phenotypes in T2DMhave been demonstrated. Zhao et al.indicated that SCFAs such as BA were significantly increased by gut microbiota and alleviated metabolic phenotype in T2DM patients after dietary fiber intervention (28). CP polysaccharides alleviated T2DM symptoms by increasing the SCFA-producing bacteria in type 2 diabetic rat models (15). Consistently, our study showed that total SCFAs, PA and BA were significantly enriched in the CP group at the end of the intervention. The mechanism responsible for the beneficial effects of SCFAs on T2DM involves the combination of SCFAs with G protein-coupled receptor 41 (GLPR41) and GLPR43 expressed by pancreatic β-cells (38, 39).

The results of BAs profiling indicated that the regulation of BA signal might be involved in the effect of CP on T2DM patients. On Day 84 of intervention, unconjugated BAs (CDCA, 12-KLCA and β-MCA) levels were increased, while conjugated BAs (GCDCA and TDCA) levels were reduced in the CP group compared with Day 0. Mantovani et al. reported that T2DM was significantly associated with the higher levels of conjugated BAs (TCDCA, TDCA, GCDCA, GDCA and GCA) as well as the lower level of unconjugated BAs (CA) (40). The report by Zhao showed that radix scutellariae water extract ameliorated hyperglycaemia, hyperlipidaemia and liver and kidney damage by significantly decreasing the contents of conjugated BAs (GDCA, GLCA, TLCA and TUDCA) in T2DM rats (41). The above findings supported our results. BAs are conformed to be involved in regulation of glucose and lipid response. BAs can regulate the gene expression of glucose, lipid synthesis and metabolism via binding with farnesoid X receptor and Gprotein-coupled bile acid receptor 5in the enterohepatic circulation (37).

Collectively, our results indicate that CP increases the levels of SCFAs and unconjugated BAs and decreases the levels of conjugated BAs, thus leading to the improvements in HbA1c and other metabolic indicators.

At last, FMT in pseudo-sterile mice was conducted to confirm the causal relationship between the gut microbiota and the effect of CP on host glucose metabolism. The results showed that glucose metabolism parameters (FBG and OGTT glucose AUC) had a significant improvement in the mice transplanted with the post-intervention microbiota from the CP group compared with the mice transplanted with pre-intervention microbiota. Zhang et al. Reported that transplanted fecal bacteria from individuals with normal glucose tolerance altered gut microbiota composition and improved FBG, 2hPBG, TC, TG, and LDL-c in db/db mice (42). Our findings supported the above results, suggesting that better glucose metabolic parameters in mice was associated with the improvement in gut microbiota dysbiosis in the patient CP008 after CP intervention.

## Strengths and limitations

There were several strengths in our study. First, all existing studies on CP were based on animals or *in vitro* experiments and this is the first clinical RCT to reveal the effect of CP on T2DM patients. Second, we explored the potential mechanism of CP-induced metabolic phenotypes improvement. Third, a pseudo-sterile mouse experiment was performed to verify the causality between the gut microbiota and CP-induced changes in glucose metabolism. However, there were some limits to the study. First, this was a single, open-label, controlled clinical trial, and the participants and researchers were aware of the intervention. Third, the sample size of diabetes patients included in the clinical study was relatively small. In addition, germ-free mice were not used in the FMT experiment.

## Conclusion

In conclusion, this 84-day clinical randomized controlled trial in T2DM patients demonstrates that CP displays a more beneficial effect in the alleviation of T2DM-associated metabolic phenotypes than Glipizide by regulating gut microbiota and metabolites, with no significant effects on liver and kidney function. However, these results should be considered preliminary, and large-scale clinical controlled studies are required to confirm these findings.

## Data availability statement

The original contributions presented in the study are included in the article/**Supplementary Material**. Further inquiries can be directed to the corresponding author.

## Ethics statement

The studies involving human participants were reviewed and approved by ethical approval department No. KY-20181226001. The patients/participants provided their written informed consent to participate in this study. The animal study was reviewed and approved by Jinan University (20190821-02).

## Author contributions

The authors’ responsibilities were as follows—XP, SC, LuZ, YL, CW and ST contributed to the conception and design of this study. XP, SC, LuZ, YL, CW, LiZ, WC, JZ and JY were involved in the acquisition and analysis of the data. XP, LiZ and LuZ interpreted the results. XP, SC and ST drafted the manuscript. All authors read and approved the final manuscript.

## Supplementary material

Supplementary Figure 1 Business License of Chenzhou Mingrun biological products Co. LTD, Hunan, China and cyclocarya paliurus leaves extracts. **(A)** Business license. **(B)** Food production license. **(C)** List of permitted varieties of food production. **(D)** Packaging of Cyclocarya paliurus leaves extracts. **(E)** Cyclocarya paliurus leaves extracts.

Supplementary Figure 2 HPLC fingerprinting of cyclocarya paliurus leaves extracts. HPLC chromatogram of standard mixture **(A)** and cyclocarya paliurus leaves extracts **(B)**. External standard: (1) 3-caffeoylquinic acid, (2) 5-caffeoylquinic acid, (3) isoquercitrin, (4) kaempferol-3-glucoside, (5) kaempferol 3-rhamnoside, and (6) quercetin.

Supplementary Figure 3 The flow chart of participant recruitment and withdrawal.

Supplementary Figure 4 Anthropometric markers in T2DM patients. **(A-F)** The changes in the levels of weight **(A)**, Waist **(B)**, Waist-hip ratio **(C)**, BMI **(D)**, SBP **(E)** and DBP **(F)**. Data are presented as means ± SD or medians (IQRs). Paired T-test or non-parametric two-tailed Wilcoxon paired sign rank test was used for intra-group comparisons and independent T-test or non-parametric K independent Wilcoxon signed-ranks was used for comparisons of the fold changes between the two groups. CP group, cyclocarya paliurus leaves extracts group; G group, Glipizide group; SD, standard deviation; IQR, interquartile range; BMI, body mass index; SBP, systolic blood pressure; DBP, diastolic blood pressure.

Supplementary Figure 5 Liver and renal function in T2DM patients. (A-H) The changes in the levels of TBIL **(A)**, DBIL **(B)**, IBIL **(C)**, ALB **(D)**, ALT **(E)**, AST **(F)**, Scr **(G)**, BUN **(H)**. Data are presented as means ± SD or medians (IQRs). Paired T-test or non-parametric two-tailed Wilcoxon paired sign rank test was used for intra-group comparisons and independent T-test or non-parametric K independent Wilcoxon signed-ranks was used for comparisons of the fold changes between the two groups. CP group, cyclocarya paliurus leaves extracts group; G group, Glipizide group; SD, standard deviation; IQR, interquartile range; TBIL, total bilirubin; DBIL, direct bilirubin; IBIL, indirect bilirubin;ALB, albumin;ALT, Alanine aminotransferase; AST, Aspartate aminotransferase; Scr, serum creatinine; BUN, blood urea nitrogen.

## Glossary

**Table:**

T2DM	type 2 diabetes mellitus
FDA	Food and Drug Administration
SCFAs	short-chain fatty acids
CP group	cyclocarya paliurus leaves extracts group
G group	Glipizide group
FBG	fasting plasma glucose
	
METs	Metabolic Equivalent Tasks
BMI	body mass index
BW	body weight
WC	waist circumference
WHR	waist to hip ratio
SBP	systolic blood pressure
DBP	diastolic blood pressure
OGTT	oral glucose tolerance test
FINS	fasting insulin
2hPINS	2-hour post-meal insulin
FCP	fasting C- peptide, 2hPCP, 2-hour post-meal C-Peptide, the area under curve of C-peptide release test, TC, total cholesterol
TG	triglycerides
LDL-c	low-density lipoprotein cholesterol
HDL-c	high-density lipoprotein cholesterol
TBIL	the total bilirubin
DBIL	direct bilirubin
IBIL	indirect bilirubin
ALB	albumin
ALT	alanine aminotransferase
AST	aspartate aminotransferase
Scr	serum creatinine
BUN	blood urea nitrogen
FMT	fecal microbiota transplantation
NC	control group
AT	antibiotic treatment
ITT	intention-to-treat
SPSS	Statistical Package for Social Science
HPLC	high-performance liquid chromatography
Rt	retention times
UV	ultraviolet
PA	propionic acid
BA	butyric acid
AA	acetic acid
IBA	isobutyric acid
VA	valeric acid
IVA	isovaleric acid
HA	hexanoic acid
BAs	bile acids
CDCA	chenodeoxycholic acid
12-KLCA	12-ketolithocholic acid
β-MCA	β-muricholic acid
TDCA	taurodeoxycholic acid
GCDCA	glycochenodeoxycholic acid
SUR	sulphonylurea receptor
GLPR41	G protein-coupled receptor 41.
