# Supplementary figures and images for: Cyclocarya paliurus leaves extracts alleviate metabolic phenotypes in Chinese T2DM patients by modulating gut microbiota and metabolites: a clinical randomized controlled trial
Source: Front Endocrinol (Lausanne). 2023 May 24;14:1176256. doi: 10.3389/fendo.2023.1176256 (PMC10246770; doi:10.3389/fendo.2023.1176256)

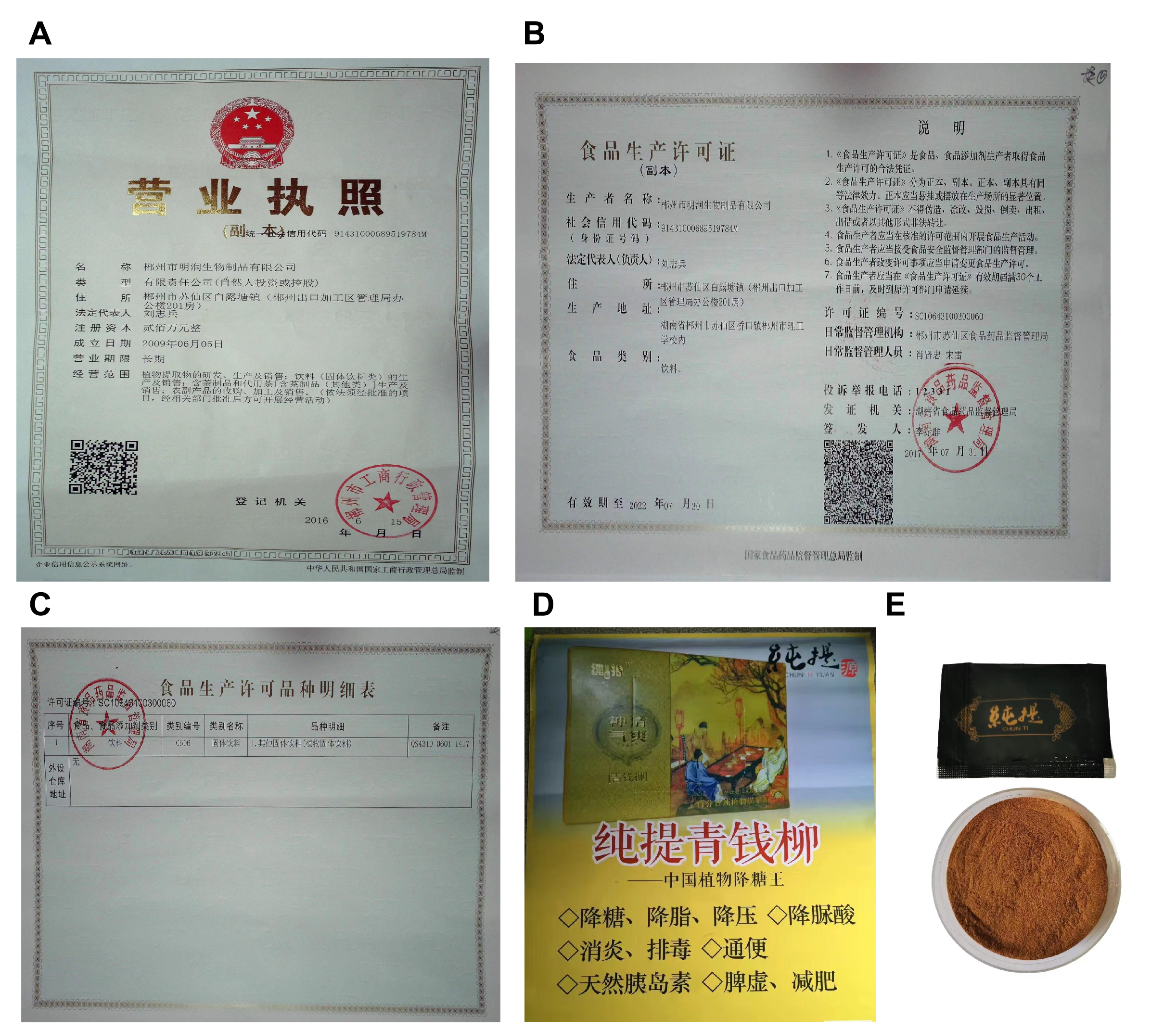

Supplement: Supplementary Figure 1 — Business License of Chenzhou Mingrun biological products Co. LTD, Hunan, China and cyclocarya paliurus leaves extracts. (A) Business license. (B) Food production license. (C) List of permitted varieties of food production. (D) Packaging of Cyclocarya paliurus leaves extracts. (E) Cyclocarya paliurus leaves extracts. [file Image_1.tif]

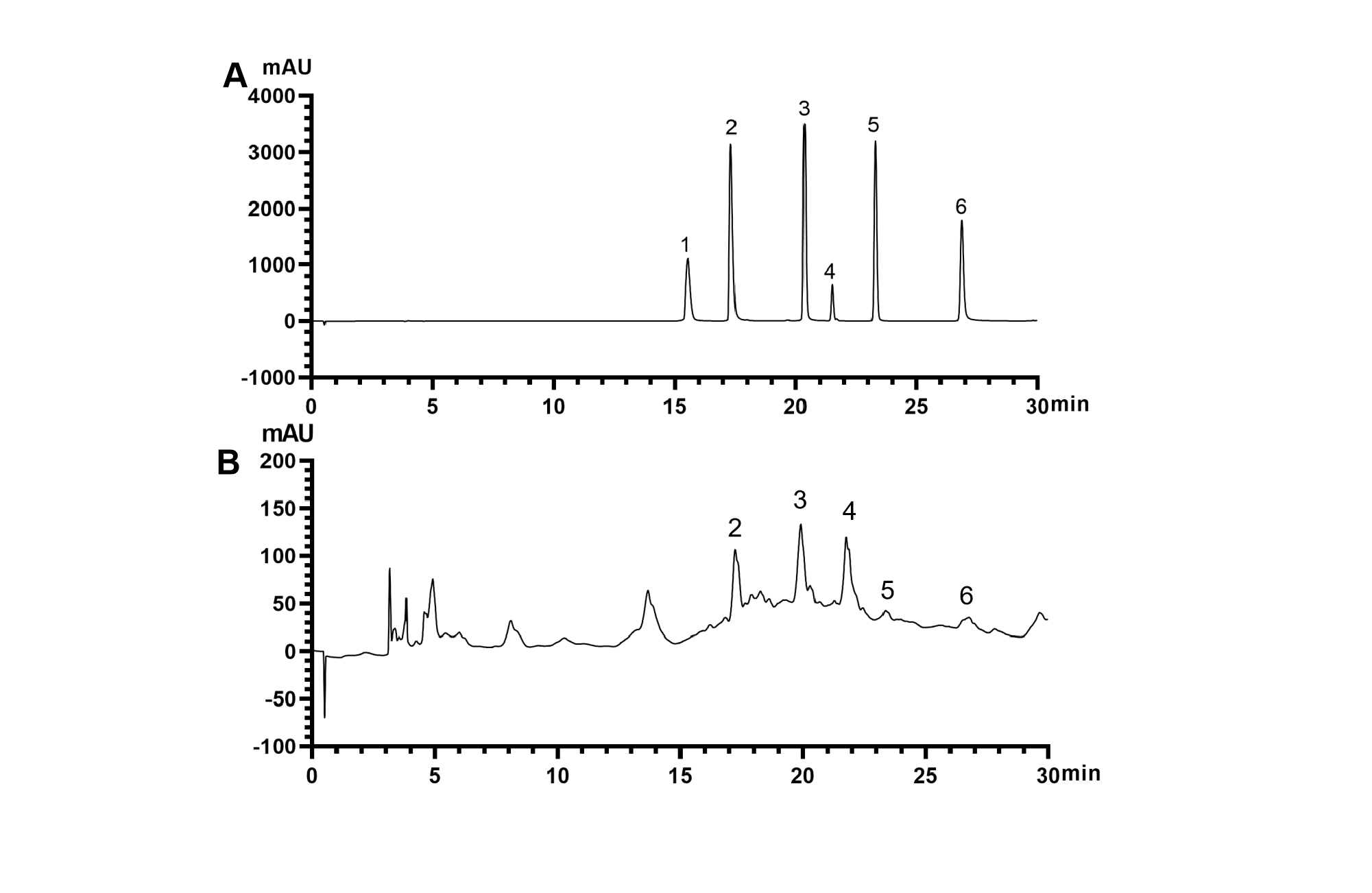

Supplement: Supplementary Figure 2 — HPLC fingerprinting of cyclocarya paliurus leaves extracts. HPLC chromatogram of standard mixture (A) and cyclocarya paliurus leaves extracts (B). External standard: (1) 3-caffeoylquinic acid, (2) 5-caffeoylquinic acid, (3) isoquercitrin, (4) kaempferol-3-glucoside, (5) kaempferol 3-rhamnoside, and (6) quercetin. [file Image_2.tif]

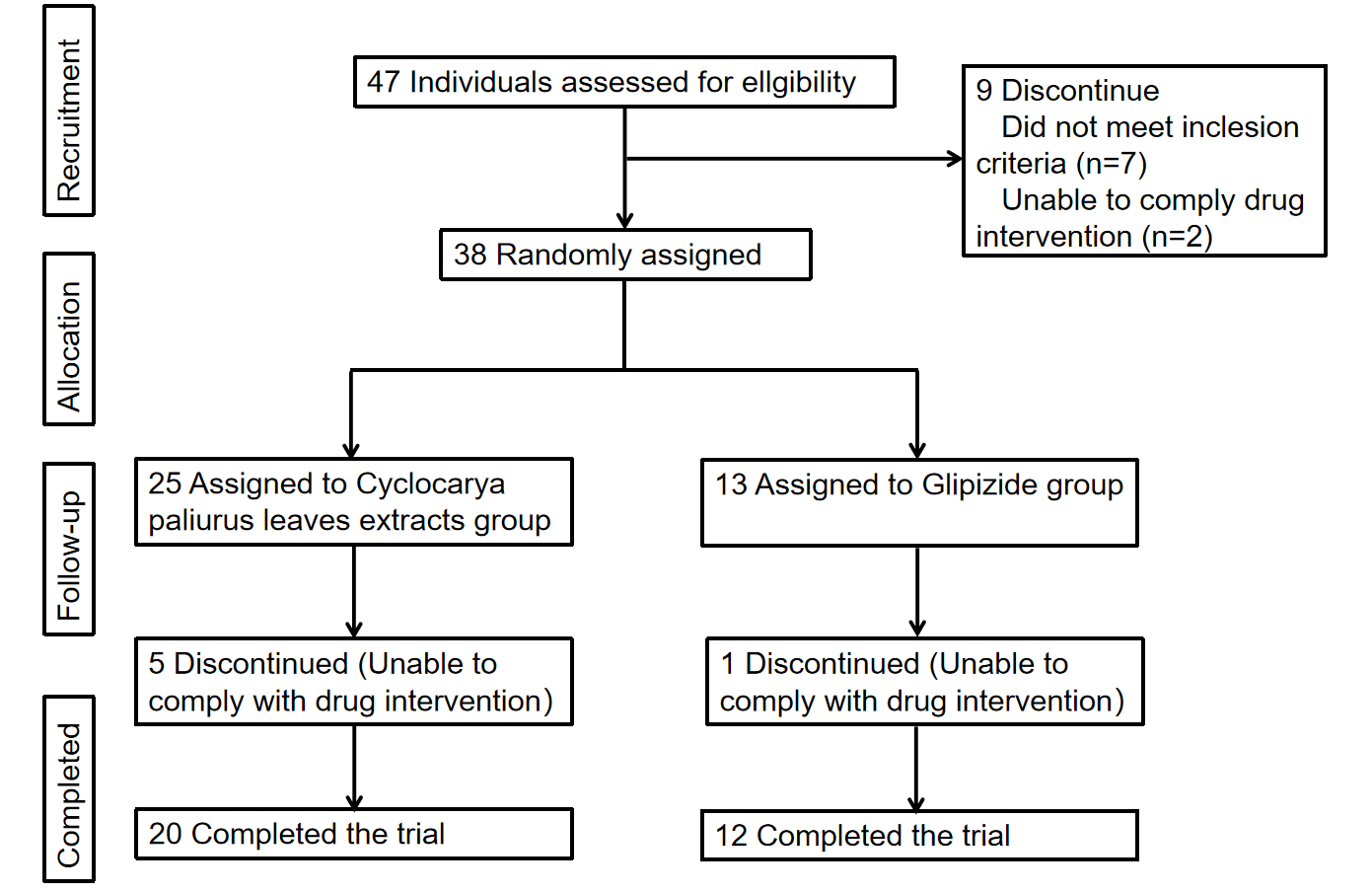

Supplement: Supplementary Figure 3 — The flow chart of participant recruitment and withdrawal. [file Image_3.tiff]

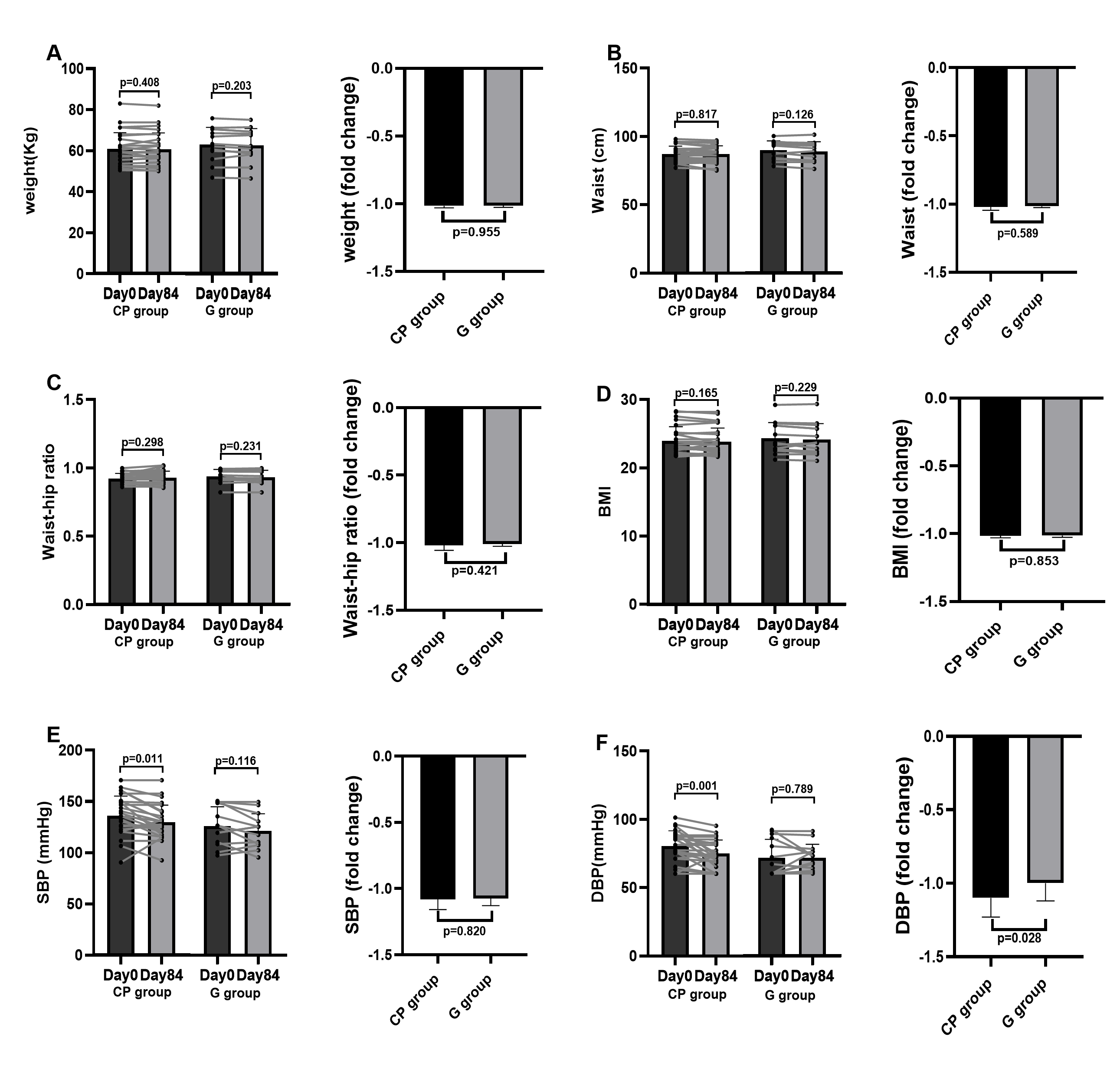

Supplement: Supplementary Figure 4 — Anthropometric markers in T2DM patients. (A-F) The changes in the levels of weight (A), Waist (B), Waist-hip ratio (C), BMI (D), SBP (E) and DBP (F). Data are presented as means ± SD or medians (IQRs). Paired T-test or non-parametric two-tailed Wilcoxon paired sign rank test was used for intra-group comparisons and independent T-test or non-parametric K independent Wilcoxon signed-ranks was used for comparisons of the fold changes between the two groups. CP group, cyclocarya paliurus leaves extracts group; G group, Glipizide group; SD, standard deviation; IQR, interquartile range; BMI, body mass index; SBP, systolic blood pressure; DBP, diastolic blood pressure. [file Image_4.tif]

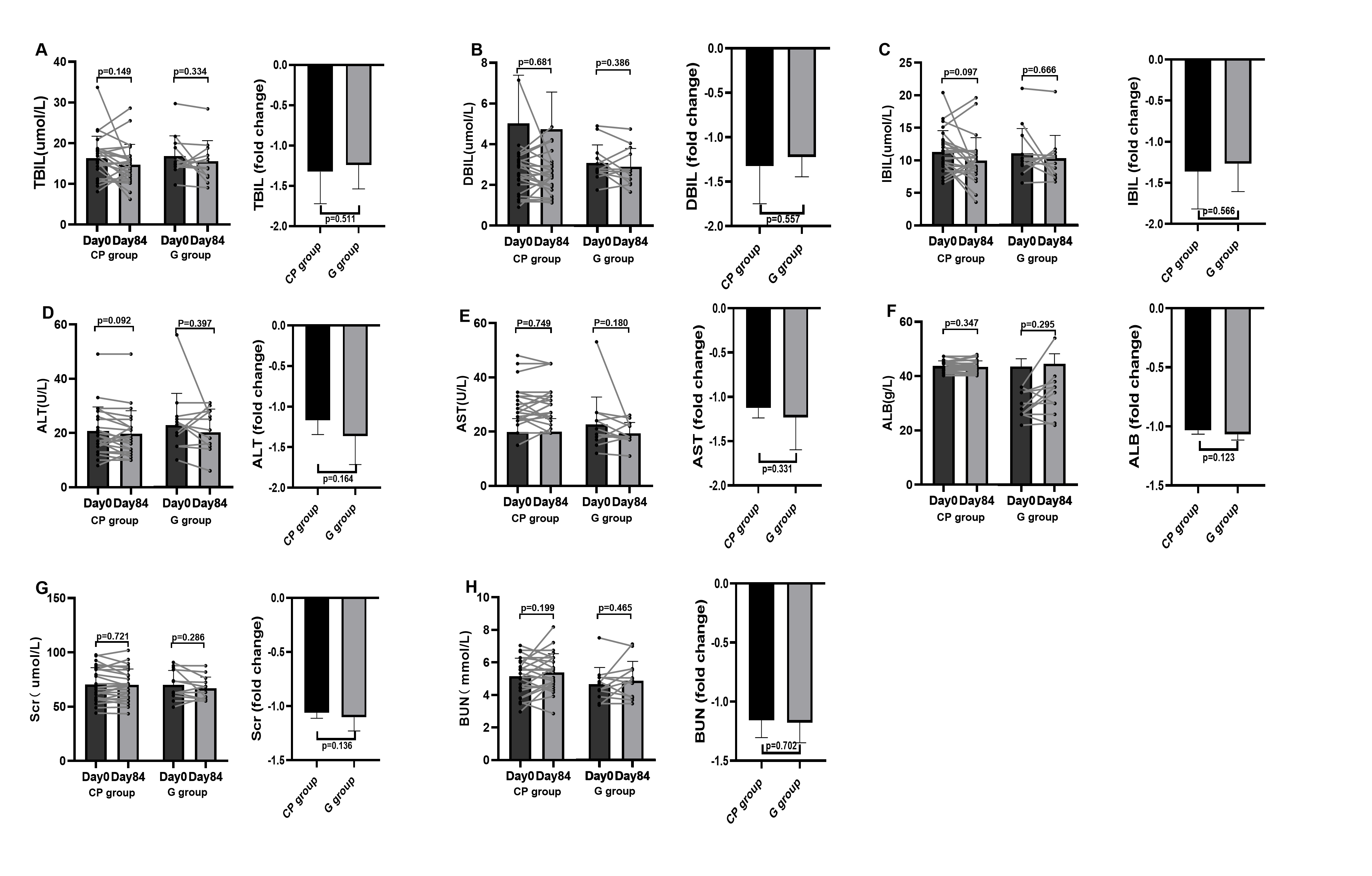

Supplement: Supplementary Figure 5 — Liver and renal function in T2DM patients. (A-H) The changes in the levels of TBIL (A), DBIL (B), IBIL (C), ALB (D), ALT (E), AST (F), Scr (G), BUN (H). Data are presented as means ± SD or medians (IQRs). Paired T-test or non-parametric two-tailed Wilcoxon paired sign rank test was used for intra-group comparisons and independent T-test or non-parametric K independent Wilcoxon signed-ranks was used for comparisons of the fold changes between the two groups. CP group, cyclocarya paliurus leaves extracts group; G group, Glipizide group; SD, standard deviation; IQR, interquartile range; TBIL, total bilirubin; DBIL, direct bilirubin; IBIL, indirect bilirubin;ALB, albumin;ALT, Alanine aminotransferase; AST, Aspartate aminotransferase; Scr, serum creatinine; BUN, blood urea nitrogen. [file Image_5.tif]
