# Supplementary material for: Cyclocarya paliurus leaves extracts alleviate metabolic phenotypes in Chinese T2DM patients by modulating gut microbiota and metabolites: a clinical randomized controlled trial
Source: Front Endocrinol (Lausanne). 2023 May 24;14:1176256. doi: 10.3389/fendo.2023.1176256 (PMC10246770; doi:10.3389/fendo.2023.1176256)
Supplement: Supplementary file 6 [file Table_1.docx]

**Supplementary Material Content 1**

Cyclocarya paliurus leaves extracts alleviate metabolic phenotypes in Chinese T2DM patients by modulating gut microbiota and metabolites: a clinical randomized controlled trial

Xiaojuan Peng^1, 2, 3#^, Sisi Chen^2#^, Lu Zhong^2#^, Yuting Li^2#^, Chutian Wu^2#^, Lixian Zhong^2^, Weiwei Chen^2^, Jinying Yang^2^, Jiahua Zeng^2^, Shaohui Tang^2^*

**Table S1. Exclusion criteria**

| Patients with evidence of T1DM, pregnant women; breast feeding women or women planning to become pregnant; |
| --- |
| Patients with evidence of severe diabetic complications (diabetic retinopathy, diabetic peripheral neuropathy, diabetic nephropathy, diabetic foot); |
| Patients with evidence of severe liver disease (chronic active viral hepatitis B or C, cirrhosis, liver cancer, glutamic pyruvic transaminase or glutamic oxalacetic transaminase levels that were 2.5 times higher than the upper limit of normal, et al); |
| Patient who had taken antibiotics within 3 months; |
| Patients with use of wight-loss drugs more than 1 month; |
| Patients with a history of gastrointestinal surgery (except appendicitis or hernia surgery); |
| Patients with history of serious mental illness within 6 months; |
| Patients receiving medical treatment for cholecystitis, peptic ulcer, urinary tract infection, acute pyelonephritis, cystitis or hyperthyroidism; |
| Patients suffering from pituitary dysfunction; |
| Patients with serious organic diseases (cancer, coronary heart disease, myocardial infarction, stroke); |
| Patients suffering from infectious disease (tuberculosis and AIDS); |
| Patients with history of alcoholism; |
| Patients with use of drugs or food that may affect intestinal flora. |

**Table S2. Phytochemical compositions of the CP**

| Components | **Content (mg/g)** |
| --- | --- |
| 3-caffeoylquinic acid | 53.4 |
| Isoquercitrin | 42.5 |
| Kaempferol-3-glucoside | 92.6 |
| Kaempferol -3-rhamnoside | 4.8 |
| Quercetin | 2.1 |

CP, cyclocarya paliurus leaves extracts.

**Table S3. Comparison of clinical parameters at baseline in T2DM participants**

| **Clinical characteristics parameters** | **CP group** | **G group** | ***P*** |
| --- | --- | --- | --- |
| **Anthropometric markers** | | | |
| BW (kg) | 60.88±7.95 | 63.10±8.36 | 0.428 |
| WC (cm) | 87.16±5.74 | 89.88±6.80 | 0.201 |
| WHR | 0.92±0.38 | 0.94±0.50 | 0.201 |
| BMI | 23.95±2.07 | 24.38±2.33 | 0.573 |
| SBP (mmHg) | 136.08±19.23 | 125.84±19.13 | 0.128 |
| DBP (mmHg) | 81.00 (73.00, 88.50) | 67.00 (60.00, 87.50) | 0.075 |
| **Blood glucose homeostasis** | | | |
| HbA1c (%) | 7.29 (7.07, 7.63) | 7.14 (7.08, 8.44) | 0.963 |
| FBG (mmol/L) | 8.37±2.05 | 7.80±1.26 | 0.548 |
| 2hPBG (mmol/L) | 14.56 (12.47, 16.58) | 14.45 (12.6, 19.00) | 0.691 |
| FINS (μIU/mL) | 6.35 (4.39, 10.93) | 4.46 (3.40, 9.80) | 0.348 |
| 2hPINS (μIU/mL) | 37.34 (27.02, 85.43） | 26.77 (21.56, 51.74) | 0.275 |
| HOMA-IR | 2.26 (1.65, 4.45) | 1.46 (1.24, 3.18) | 0.261 |
| Fasting C-Peptide (ng/mL) | 1.11 (0.88, 1.73) | 0.99 (0.85, 1.30) | 0.432 |
| 2hPCP (ng/mL) | 3.82 (3.15, 5.11) | 3.53 (2.83, 4.40) | 0.451 |
| OGTT glucose AUC (mmol/L/min) | 2226.15 (1972.72, 2688.37) | 2258.10 (2026.90, 2827.05) | 0.590 |
| Insulin release test AUC (μIU/mL/min) | 4942.2 (3613.35, 10822.2) | 4249.2 (2807.7, 5489.25） | 0.258 |
| C-Peptide release test AUC (ng/mL/min) | 541.8 (419.6, 711.8) | 420.75 (378.45, 634.20) | 0.332 |
| **Blood lipid homeostasis** | | | |
| TG (mmol/L) | 2.38±1.51 | 1.67±0.92 | 0.129 |
| TC (mmol/L) | 5.65±1.35 | 4.54±0.89 | 0.112 |
| LDL-c (mmol/L) | 3.40±1.24 | 2.55±0.71 | 0.328 |
| HDL-c (mmol/L) | 1.17±0.42 | 1.22±0.32 | 0.680 |
| **Liver and renal function** | | | |
| TBIL (umol/L) | 16.34±5.38 | 16.82±5.00 | 0.792 |
| DBIL (umol/L) | 4.8 (3.35, 6.05) | 5.2 (4.95, 6.45) | 0.242 |
| IBIL (umol/L) | 11.0 (9.15, 13.00) | 9.90 (9.20, 12.35) | 0.590 |
| ALB (g/L) | 43.73±1.91 | 43.53±2.88 | 0.798 |
| ALT (U/L) | 19.00 (15.00, 27.00) | 23.00 (15.00, 25.50) | 0.677 |
| AST (U/L) | 19.00 (16.50, 22.50) | 19.0 (17.50, 25.50) | 0.413 |
| Scr (umol/L) | 70.47±15.51 | 69.90±13.44 | 0.912 |
| BUN (mmol/L) | 5.15±1.10 | 4.66±1.03 | 0.196 |

The data are shown as the mean ± SD for normal variables or median (IQR) for non-normal variables. Independent T-test and non-parametric K independent Wilcoxon signed-ranks was used for comparisons at baseline between the two groups.

CP group, cyclocarya paliurus leaves extracts group; G group, Glipizide group; SD, standard deviation; IQR, interquartile range; BW, body weight; WC, waist circumference; WHR, waist to hip ratio; BMI, body mass index; SBP, systolic blood pressure; DBP, diastolic blood pressure; HbA1c, hemoglobin A1c; FBG, fasting blood glucose; 2hPBG, 2-hour post-meal blood glucose; FINS, fasting insulin; 2hPINS, 2-hour post-meal insulin; HOMA-IR, homeostasis model assessment for insulin resistance = (fasting blood glucose×fasting insulin/ 22.5); 2hPCP, 2-hour post-meal C-Peptide; OGTT, oral glucose tolerance test; AUC, area under curve; TG, triglycerides; TC, total cholesterol; LDL-c, low-density lipoprotin cholesterol; HDL-c, high-density lipoprotein cholesterol; TBIL, total bilirubin; DBIL, direct bilirubin; IBIL, indirect bilirubin; ALB, albumin; ALT, alanine aminotransferase; AST, aspartate aminotransferase; Scr, serum creatinine; BUN, blood urea nitrogen.

**Table S4.** Physical activity energy expenditure (MET-h/week) in T2DM patients.

| **Day** | **CP group (n=25)** | **G group (n=13)** | **P** |
| --- | --- | --- | --- |
| 0 | 154.70 (136.23, 171.50) ^&^ | 155.29 (147.18, 174.93) ^&^ | 0.633 |
| 42 | 153.86 (142.42, 183.61)^&^ | 161.98 (150.12, 176.75)^&^ | 0.528 |
| 84 | 159.01 (135.39, 185.89)^&^ | 157.01 (141.07, 178.06)^&^ | 0.841 |

The data are shown as median (IQR). ^&^P﹥0.05, comparison among Day 42, Day 84 and Day 0 at the same group. P, comparison between CP group and G group at the same time point. CP group, cyclocarya paliurus leaves extracts group; G group, Glipizide group; SD, standard deviation; IQR, interquartile range; MET, metabolic equivalent tasks.

The calculation formula of physical activity energy consumption is as follows: physical activity energy expenditure (MET-h/week) = MET level × activity time (hour) × times per week. For example, a participant in the F group slept 9 hours a day, 7 days a week; Watch TV/computer/listen to music 4 hours, 7 days a week; Dinning 40 minutes, 7 days a week; Work 8 hours, 5 days a week; Walk 30 minutes, 7 days a week; 40 minutes by car, 7 days a week; Do 30 minutes of housework, three times a week; 30 minutes of running, once a week. The total MET-h/week = (0.9 × 9 h/time × 7 times/week) + (1.0 × 4 h/time × 7 times/week) + (1.5 × 0.7 h/time × 7 times/week) + (1.5 × 8 h/time × 5 times/week) + (3.0 × 0.5 h/time × 7 times/week) + (1.0 × 0.7 h/time × 7 times/week) + (1.5 × 0.5 h/time × 3 times/week) + (7.0 × 0.5 h/time × 1 times/week) = 173.2 MET-h/week.

**Table S5. Proportion of T2DM patients with HbA1c﹤7%.**

| **Item** | **CP group** | | | **G group** | | |
| --- | --- | --- | --- | --- | --- | --- |
|  | **Day 0 (n=25)** | **Day 84 (n=25)** | ***P-value*** | **Day 0 (n=13)** | **Day 84 (n=13)** | ***P-value*** |
| The proportion of subjects with HbA1c﹤7%, % | 16.00 | 56.00 | 0.002 | 15.40 | 46.10 | 0.038 |

CP group, cyclocarya paliurus leaves extracts group; G group, Glipizide group; HbA1c, hemoglobin A1

**Table S6.** Comparison of the abundances of the gut microbiota at the phylum level.

| **Phylum (%)** | **CP group** | | | **G group** | | | | ***PP*** |
| --- | --- | --- | --- | --- | --- | --- | --- | --- |
|  | **Day 0 (n=25)** | **Day 84 (n=25)** | **P-value** | **Day 0 (n=13)** | | **Day 84 (n=13)** | **P-value** |  |
| Firmicutes | 56.4±12.37 | 52.87±16.07 | 0.246 | 30.84±18.06 | | 40.99±19.94 | 0.841 | 0.009 |
| Bacteroidetes | 34.07±14.84 | 35.03±20.16 | 0.761 | 41.79±20.31 | | 42.85±20.42 | 0.849 | 0.178 |
| Proteobacteria | 4.56 (1.84, 6.38) | 3.06 (1.66, 7.43) | 0.399 | 1.21 (0.02, 5.65) | | 0.19 (0.01, 4.92) | 0.857 | 0.361 |
| Actinobacteria | 0.80 (0.26, 1.53) | 0.78 (0.32, 0.98) | 0.455 | 0.24 (0.09, 0.47) | | 0.22 (0.11, 1.53) | 0.583 | 0.077 |
| Verrucomicrobia | 0.03 (0.01, 0.19) | 0.11 (0.02, 2.23) | 0.004 | 0.86 (0.18, 7.14) | 0.19 (0.01, 1.60) | | 0.433 | 0.271 |
| Fusobacteria | 0.004 (0.00, 0.06） | 0.01 (0.00, 0.07) | 0.227 | 0.30 (0.01, 4.92) | | 1.00 (0.01, 5.19) | 0.754 | 0.014 |
| Synergistetes | 0.04 (0.00, 0.14) | 0.01 (0.00, 0.07) | 0.227 | 0.00 (0.00, 0.06) | | 0.00 (0.00, 0.09) | 0.272 | 0.951 |
| Cyanobacteria | 0.01 (0.00, 0.35) | 0.01 (0.00, 0.05) | 0.940 | 0.00 (0.00, 0.02) | | 0.00 (0.00, 0.13) | 1.000 | 0.190 |
| Patescibacteria | 0.02±0.03 | 0.03±0.04 | 0.058 | 0.01±0.01 | | 0.01±0.01 | 0.481 | 0.259 |

The data are shown as the mean ± SD for normal variables or median (IQR) for non-normal variables. Paired T-test or non-parametric two-tailed Wilcoxon paired sign rank test was used for intragroup comparisons. CP group, cyclocarya paliurus leaves extracts group; G group, Glipizide group; SD, standard deviation; IQR, interquartile range.

P, comparison of the baseline between the CP group and the G group.

**Table S7.** Comparison of the abundances of the gut microbiota at the genus level.

| **genus (%)** | **CP group** | | | **G group** | | | ***PP*** |
| --- | --- | --- | --- | --- | --- | --- | --- |
|  | **Day 0 (n=25)** | **Day 84 (n=25)** | **P-value** | **Day 0 (n=13)** | **Day 84 (n=13)** | **P-value** |  |
| Bacteroides | 26.19±12.42 | 25.12±1418 | 0.676 | 26.47±17.90 | 24.01±16.55 | 0.583 | 0.955 |
| Prevotella_9 | 15.41±16.89 | 7.17±5.45 | 0.030 | 24.30±20.37 | 29.42±23.85 | 0.330 | 0.319 |
| Faecalibacterium | 4.09±4.47 | 6.37±5.19 | 0.010 | 4.86±4.87 | 5.88±6.22 | 0.540 | 0.627 |
| Akkermansia | 0.08 (0.01, 1.45) | 1.34 (0.10, 6.00) | 0.010 | 1.88 (0.02, 3.76) | 1.78 (0.08, 8.56) | 0.530 | 0.168 |
| Phascolarctobacteriu | 6.39 (0.74, 9.54) | 7.06 (1.06, 10.55) | 0.823 | 1.45 (0.20, 5.43) | 1.54 (0.11, 5.26) | 0.437 | 0.104 |
| Ruminococcaceae_UCG-002 | 1.10 (0.45, 6.47) | 1.18 (0.10, 6.73) | 0.654 | 0.46 (0.07, 6.24) | 0.36 (0.04, 1.12) | 0.071 | 0.286 |
| Klebsiella | 0.85 (0.07, 3.57) | 0.56 (0.04, 5.23) | 0.550 | 0.49 (0.07, 6.31) | 0.07 (0.04, 6.75) | 0.433 | 0.761 |
| Dialister | 3.62 (0.12, 7.49) | 3.67 (0.08, 6.08) | 0.823 | 0.78 (0.04, 2.97) | 0.78 (0.32, 4.80) | 0.638 | 0.300 |
| Megamonas | 0.07 (0.01, 5.00) | 0.05 (0.02, 8.23） | 0.296 | 0.46 (0.10, 4.25) | 0.03 (0.01, 1.72) | 0.584 | 0.952 |
| Lachnoclostridium | 1.82 (0.32, 7.00) | 2.09 (0.45, 6.25) | 0.823 | 2.12 (0.22, 5.01) | 1.86 (0.28, 3.41) | 0.695 | 0.649 |

The data are shown as the mean ± SD for normal variables or median (IQR) for non-normal variables. Paired T-test or non-parametric two-tailed Wilcoxon paired sign rank test was used for intragroup comparisons. CP group, cyclocarya paliurus leaves extracts group; G group, Glipizide group; SD, standard deviation; IQR, interquartile range.

P, comparison of the baseline between the CP group and the G group.

**Table S8. Comparison of the** **SCFAs related to the gut microbiome.**

| **SCFAs (mg/g dry feces)** | **CP group** | | | **G group** | | | ***PP*** |
| --- | --- | --- | --- | --- | --- | --- | --- |
|  | **Day 0 (n=25)** | **Day 84 (n=25)** | **P-value** | **Day 0 (n=13)** | **Day 84 (n=13)** | **P-value** |  |
| Total SCFAs | 5.50±2.76 | 6.49±2.72 | 0.006 | 6.30±2.89 | 6.96±2.14 | 0.304 | 0.413 |
| AA | 2.26±1.49 | 2.27±1.38 | 0.930 | 2.96±1.42 | 3.25±1.46 | 0.305 | 0.173 |
| PA | 1.55±1.29 | 1.82±1.28 | 0.032 | 1.65±0.93 | 1.79±0.62 | 0.560 | 0.841 |
| BA | 1.05±0.83 | 1.58±1.45 | 0.027 | 1.21±0.91 | 1.27±0.76 | 0.756 | 0.579 |
| IBA | 0.11 (0.06, 0.39) | 0.17 (0.09, 0.41) | 0.191 | 0.09 (0.05, 0.21) | 0.13 (0.06, 0.43) | 0.875 | 0.584 |
| VA | 0.10 (0.02, 0.35) | 0.18 (0.06, 0.41) | 0.023 | 0.12 (0.01, 0.13) | 0.10 (0.04, 0.16) | 0.209 | 0.300 |
| IVA | 0.17±0.16 | 0.21±0.25 | 0.253 | 0.10±0.10 | 0.15±0.20 | 0.238 | 0.174 |
| HA | 0.00 (0.01, 0.07) | 0.01 (0.00, 0.01) | 0.765 | 0.01 (0.00, 0.10) | 0.00 (0.00, 0.16) | 0.347 | 0.627 |

The data are shown as the mean ± SD for normal variables or median (IQR) for non-normal variables. Paired T-test or non-parametric two-tailed Wilcoxon paired sign rank test was used for intra-group comparisons. P, comparison of the baseline between the CP group and the G group. CP group, cyclocarya paliurus leaves extracts group; G group, Glipizide group; SD, standard deviation; IQR, interquartile range; SCFAs, short chain fatty acids; AA, acetic acid; PA, propionic acid; BA, butyric acid; IBA, isobutyric acid; VA, valeric acid; IVA, isovalerlc acid; HA, hexanoic acid

**Table S9. Comparison of the BAs related to the gut microbiome**

| **BAs**  **(ng/g dry feces)** | **CP group** | | | **G group** | | | ***PP*** |
| --- | --- | --- | --- | --- | --- | --- | --- |
|  | **Day 0 (n=25)** | **Day 84 (n=25)** | **P-value** | **Day 0 (n=13)** | **Day 84 (n=13)** | **P-value** |  |
| **Unconjugated BAs** | | | | | | |  |
| CDCA | 83793.8±73182.9 | 115392.6±100777.1 | 0.006 | 142313.9±76341.7 | 124199.7±79808.5 | 0.304 | 0.028 |
| DCA | 46061.1±38432.1 | 49313.4±41709.7 | 0.273 | 66653.5±28924.4 | 69856.8±41206.9 | 0.590 | 0.099 |
| LCA | 42941.49±26494.8 | 44695.89±24453.42 | 0.690 | 35381.12±16271.5 | 47969.6±29703.88 | 0.248 | 0.355 |
| UDCA | 39592.51±46906.14 | 48485.43±54050.31 | 0.233 | 56844.03±36679.16 | 69032.81±45672.55 | 0.235 | 0.257 |
| 7-KLCA | 9021.56 (2810.72, 15943.96) | 8165.53 (2083.63, 18066.81) | 0.627 | 13931.55 (9643.05, 43388.09) | 15307.15 (7811.13, 40491.15) | 0.814 | 0.038 |
| CA | 5309.14±5449.60 | 5910.53±6193.52 | 0.228 | 12042.90±12337.56 | 12465.4±14360.91 | 0.733 | 0.081 |
| 12-KLCA | 9878.39±5211.01 | 12773.43±6752.90 | 0.035 | 10533.36±5099.58 | 12806.28±8598.59 | 0.398 | 0.713 |
| β-MCA | 417.90±284.29 | 690.06±482.43 | 0.007 | 660.47±662.53 | 493.56±488.25 | 0.416 | 0.122 |
| ω-MCA | 408.38 (224.05, 802.25) | 551.02 (303.42, 1117.91) | 0.086 | 758.58 (527.76, 1977.58) | 1003.50 (456.06, 2263.73) | 0.754 | 0.085 |
| **Conjugated BAs** | | | | | | |  |
| GCDCA | 945.42 (437.76, 1950.48) | 447.21 (263.97, 1221.78) | 0.002 | 818.32 (175.92, 5742.97) | 798.23 (107.31, 4168.86) | 0.433 | 0.738 |
| GCA | 385.30 (138.80, 2353.55) | 750.12 (129.96, 2427.75) | 0.627 | 1209.25 (679.68, 3920.72) | 2157.91 (748.36, 2766.68) | 0.875 | 0.030 |
| TCA | 376.22 (78.74, 997.56) | 290.54 (88.97, 720.40) | 0.478 | 252.44 (45.97, 1597.41) | 622.59 (69.46, 1455.98) | 0.754 | 0.927 |
| TCDCA | 99.74 (36.71, 336.36) | 111.22(33.70, 313.52) | 0.526 | 323.56 (90.30, 1075.63) | 267.86 (96.99, 863.34) | 0.530 | 0.035 |
| GUDCA | 99.08 (54.89, 180.38) | 88.92 (36.16, 136.79) | 0.179 | 141.45 (38.99, 548.07) | 126.78 (46.58, 289.99) | 0.814 | 0.411 |
| GDCA | 667.59±789.56 | 620.59±516.52 | 0.805 | 771.30±687.67 | 1216.47±1584.13 | 0.191 | 0.691 |
| TUDCA | 64.16 (14.97, 131.37) | 82.39 (15.38, 170.22) | 0.654 | 56.35 (23.94, 270.34) | 31.45 (17.30, 162.07) | 0.937 | 0.584 |
| TLCA | 91.56 (37.79, 151.18） | 78.78 (28.49, 169.12) | 0.550 | 101.57 (36.55, 269.11) | 89.79 (48.22, 223.60) | 0.388 | 0.605 |
| Tβ-MCA | 79.93±69.22 | 75.16±73.83 | 0.588 | 174.86±159.48 | 173.26±165.05 | 0.393 | 0.074 |
| GLCA | 181.04±325.92 | 175.93±184.30 | 0.936 | 181.61±231.67 | 193.03±262.72 | 0.899 | 0.996 |
| TDCA | 75.22 (19.57, 210.94) | 73.86 (15.33, 131.16) | 0.004 | 98.91 (25.08, 144.72) | 71.87 (20.17, 118.27) | 0.937 | 0.832 |

The data are shown as the mean ± SD for normal variables or median (IQR) for non-normal variables. Paired T-test or non-parametric two-tailed Wilcoxon paired sign rank test was used for intragroup comparisons. P, comparison of the baseline between the CP group and the G group. CP group, cyclocarya paliurus leaves extracts group; G group, Glipizide group; SD, standard deviation; IQR, interquartile range; BA, bile acids; CDCA, chenodeoxycholic acid; DCA, deoxycholic acid; LCA, lithocholic acid; UDCA, ursodeoxycholic acid; 7-KLCA, 7-ketolithocholic acid; CA, cholic acid; 12-KLCA, 12-ketolithocholic acid; β-MCA, β-muricholic acid; ω-MCA, ω-muricholic acid; GCDCA, glycochenodeoxycholic acid; GCA, glycocholic acid; TCA, taurocholic acid; TCDCA, taurochenodeoxycholic acid; GUDCA, glycoursodeoxycholic acid; TDHCA, taurodehydrocholic acid; GDCA, glycodeoxycholic acid; TUDCA, tauroursodeoxycholic acid; TLCA, taurolithocholic acid; Tβ-MCA, tauro-β-muricholic acid; GLCA, glycolithocholic acid; TDCA, taurodeoxycholic acid.
