# Supplementary material for: Cyclocarya paliurus leaves extracts alleviate metabolic phenotypes in Chinese T2DM patients by modulating gut microbiota and metabolites: a clinical randomized controlled trial
Source: Front Endocrinol (Lausanne). 2023 May 24;14:1176256. doi: 10.3389/fendo.2023.1176256 (PMC10246770; doi:10.3389/fendo.2023.1176256)
Supplement: Supplementary file 7 [file Table_2.docx]

**Supplementary Material Content 2**

**Cyclocarya paliurus leaves extracts alleviate metabolic phenotypes in Chinese T2DM patients by modulating** **gut microbiota and metabolites: a clinical randomized controlled trial**

Xiaojuan Peng^1, 2, 3#^, Sisi Chen^2#^, Lu Zhong^2#^, Yuting Li^2#^, Chutian Wu^2#^, Lixian Zhong^2^, Weiwei Chen^2^, Jinying Yang^2^, Jiahua Zeng^2^, Shaohui Tang^2^*

**Supplementary Methods**

**Physical activity (PA) assessment**

On day 0, day 42 and day 84 of the intervention, the patients were requested to finish daily physical activity questionnaire using the 7-day recall method. The questionnaire included 19 types: after-work activities (7 types), activities at work (5 types), other daily activities (3 types), and physical activities (4 types). The researchers calculated the cumulative daily time of participants’ daily life, work and other daily activities, and the time of irregular activities (times per week and cumulative time per time) converted to daily time. Metabolic Equivalent Tasks (MET) was used to calculate the energy expenditure of PA activities. MET is defined as the ratio of energy expenditure during the activity to resting metabolic rate [1]. According to the compendium of physical activities, each type of activity has a metabolic equivalent task (MET) score [2]. Total PA was expressed in MET hour per week (MET-h∙week^-1^). The total PA MET-h∙week^-1^ = sum of after-work activities + activities at work + other daily activities + physical activities MET-h∙week^-1^ scores. Physical activity items and MET values listed in Table e1.

**Table e1. Physical activity items and MET values**

| **Physical activity items (hour)** | **MET value** |
| --- | --- |
| **After-work activities (including activities after retirement)** |  |
| Sleep (including nap time and awake time) | 0.9 |
| Dining | 1.5 |
| Use the computer | 1.5 |
| Watching TV | 1.0 |
| Sit for rest or recreation (chess, poker or mahjong, watching TV, listening to music, playing an instrument, etc.) | 1.0 |
| Housework or other standing activities (cooking, washing dishes, bathing, washing laundry, babysitting, waiting for the bus, etc.) | 1.5 |
| Walking (commuting to work, shopping, go to the parks, etc.) | 3.0 |
| **Activities at work (including part-time work after retirement)** |  |
| Sit down (reading, writing, doing office work on a computer, etc.) | 1.5 |
| Light work (selling, giving lectures, etc.) | 2.3 |
| Medium and heavy manual work (cleaning, construction, mechanical operation, etc.) | 3.5 |
| Light physical work, need to walk but no weight (<5 kg) | 3.3 |
| Walking, heavy physical work, need to carry weight, or physical strength for a long time | 8.0 |
| **Other daily activities** |  |
| Ride on a bicycle | 4.0 |
| Take a ride or drive | 1.0 |
| Climb the stairs | 3.5 |
| **Physical activity** |  |
| Ball games (badminton, table tennis, tennis, basketball, volleyball, etc.) | 4.0 |
| Athletic events (swimming, running and other athletics events) | 7.0 |
| Aerobics, dancing and other aerobics, tai chi, qigong, yoga etc. | 6.0 |
| Other physical activity of moderate intensity | 6.0 |

**Gut microbiome analysis**

**Collection and storage** On Day 0 and Day 84, Participants followed standard procedures to collect fecal samples from toilets near the laboratory and placed them into 10ml sterile cryopreservation tubes, with 3 tubes for each subject (no less than 5 grams each tube). The fecal samples were immediately stored in a -80℃ freezer until freeze-drying procedure and analyzed.

**DNA extraction of intestinal flora** After drying the stool sample, a grinding chamber was used to grind the samples. Then DNA was extracted from fecal sample using the E.Z.N.A.® Stool DNA Kit (Avantor, USA). The purity of DNA samples was detected by NanoDrop microspectrophotometer, and the integrity of DNA samples was detected by 2% agarose gel electrophoresis.

**Gut microbiota analysis** The DNA was amplified and purified by PCR, and the V3-V4 region of 16S rRNA gene was enriched with specific primers. The primers were F (5 '-GTGCCAGCMGCCGCGG-3 ') and R (5' -CCGTCAattCMTTTRAGTTT-3 '). The purified amplified product was connected with the sequencing adapter to construct the sequencing library. Genome sequencing was conducted on the Lumina HiSeg 2500 platform.

**SCFAs analysis**

Adding 1ml 0.5% phosphoric acid solution to 20mg freeze-dried stool sample for grinding (ball mill MM400 for 10s at 20Hz, Retsch, Germany), vortex mixing (10min, MIX-200, China), and ultrasonic treatment (5 minutes, incubated in ice water). Samples were centrifuged at 12000rpm/min at 4℃ for 10min, and supernatant (100 μ L) was placed in centrifuge tube, then added with 500ul MTBE (containing internal standard) solution and then treated by ultrasound for 5 min (incubated in ice water) and centrifuged for 10 min at 12000 r/min at 4°C. After centrifugation, 200μL of supernatant was absorbed into the sample bottle and stored in the refrigerator at -20℃ for detection by 7890B-7000D Gas chromatograph-Mass Spectrometer system (Agilent, CA).

**BAs analysis**

20 mg of freeze-dried fecal samples were extracted with 200 μL methanol. After vibrating at 2500 rpm for 10min, the mixture was transferred to a -20℃ refrigerator for 10min to precipitate protein. After centrifugation at 12000 r/min for 10 min, the supernatant was evaporated to dryness. Then, the extracts was reconstituted in 100 μL 50% methanol (V/V) and submitted to LC-MS analysis. BA measurement was performed by the QTRAP 6500+ LC-MS/MS system (SCIEX, Canada).

**F****ecal microbiota transplantation (FMT) in pseudo-sterile mice**

**Preparation of pseudo-sterile mice model** A total of 50 C57BL/6J mice (weight, 25.00±2g; age, 10 weeks) were purchased from SiPeiFu Biotechnology Company Limited (Beijing, China) and approved by the experiment animal ethics committee of Jinan University (20190821-02). The mice were raised in the specific pathogen-free animal experiment center in Jinan University, with constant temperature (24 ±2℃), constant humidity 55±10% and a 12-h light/dark cycle. After adaptive feeding for 10 days, the mice were randomly divided into a normal control group (NC, n=10) and an antibiotic treatment group (AT, n=40). To deplete the gut microbiota and prepare pseudo germ-free mice, the mouse in antibiotic treatment group (AT) were given sterile water containing four antibiotics, with concentrations of ampicillin (1g/L, Aladdin, CAS A105483), neomycin sulfate (1g/L, Aladdin, CAS N109017), metronidazole (1g/L, Aladdin, CAS M109874) and vancomycin (500mg/L, Aladdin, CAS V105495) ^5^ and normal chow for 4 weeks. Sterile water containing antibiotics was changed every two days. Mice in normal control group were fed with normal sterile water and normal chow. All mice were fed freely and weighed once a week. At the end of antibiotics treatment, mice feces were collected in a sterile environment, placed in sterile cryopreservation tubes, and stored in a -80℃ refrigerator for gut microbiome analysis. The detection of intestinal flora has been described above.

**Preparation of** **fecal fungus suspension** Among the participants with significant changes in gut microbiota in the CP group, the fecal samples at Day 0 and Day 84 of one participant were randomly selected for transplantation. Moreover, the fecal samples at Day 0 and Day 84 of one participant in the G group were also randomly selected for transplantation. Frozen stool specimens were thawed at room temperature (25℃) and placed in a biosafety cabinet (80%N2∶10%CO2∶10%H2, Biobase). Dissolve 1g fecal material in a ratio of 12.5 mL sterile normal saline, agitate the feces with sterile glass beads (2mm in diameter) for 5 minutes and undergo gravity settlement for 5 minutes. About 20mL fecal bacteria suspension was obtained by mixing the supernatant with an equal amount of sterile normal saline of 30% glycerol. The suspension was divided into anaerobic tubes (FM Scientific No. 2651-6001), each tube was 1.2mL, and finally placed in a refrigerator at -80℃ for use.

**Fecal microbiota transplantation** A total of 40 pseudo germ-free mice were randomly divided into four groups. Fecal samples were collected from two participants (CP008 from the CP group and G006 from the G group) at Day 0 and Day 84. Then the four groups of mice were oral gavaged with 200µL of one of the following fecal suspension inocula CP008 at Day 0 (CP-pre; n=10), CP008 at Day 84 (CP-post; n=10), G006 at Day 0 (G-pre; n=10), and G006 at Day 84 (G-post; n=10). Gavage was administered every two days for 14 days.

**Oral glucose tolerance test (OGTT)** OGTT was performed on day 14 of fecal bacteria transplantation. After fasting for 8h, fasting blood glucose (FBG) was measured. After measuring the FBG, the body weight was weighed immediately, and 20% glucose was given at 2g glucose per kg body weight (2g/kg) by gavage. Blood glucose levels were measured at 30min, 60min, 90min and 120min after gavage.

**Diabetic education**

Dietary guidance brochures (electronic version) were prepared and distributed to each enrolled participant for diabetes and nutritional education based on China guideline for type 2 diabetes in 2017 [3].

**Justification**

It has been shown that C. paliurus leaves extracts can reduce blood glucose and relieve the symptoms of T2DM in T2DM mice or T2DM rats [4, 5]. However, to our knowledge, no existing literature or research is available to address the question concerning whether C. paliurus leaves extracts can improve glucose and lipid metabolism in T2DM patients. Thus, the effects of C. paliurus leaves extracts on HbA1c and other metabolic phenotypes in T2DM patients should first be tested in smaller RCTs before large RCTs are designed.

**Potential risks**

Before starting the RCT, we have considered the potential risk. However, the risk of our study is relatively small. The potential risks are as follows.

1. Potential risk of C. paliurus leaves extracts (CP): It has been reported that CP can alleviate T2DM symptoms in T2DM mice or T2DM rats and no adverse events are reported [4, 5]. Though we designed a CP group in our study, the intervention period was only 84 days and might not have a long-term effect on T2DM patients. During the intervention, all participants were required to monitor FBG and 2hPBG every day. Once FBG was more than 12.5 mmol/L, the participants will receive the Glipizide in addition to the CP. Thus, the potential risk of CP was very small.
2. Blood drawing：The blood was drawn by experienced nurses, so there was little risk for blood drawing.
3. Blood loss: During the entire intervention period, peripheral blood was extracted only twice, 15ml each time. Thus, there was no risk for blood loss.

**Potential benefits**

At present, anti-diabetic medications have many unwanted side effects such as lactic acidosis and hypoglycemia, and ultimately fail to control blood glucose levels [6]. It has been reported that C. paliurus can reduce the levels of blood glucose, blood lipid and blood pressure in rats and no adverse events were reported[4, 5], suggesting C. paliurus may be beneficial effects on T2DM patients. At the end of the study, subjects would receive a copy of the results, which will be interpreted by a professional researcher. After the study, subjects may have an additional choice in the treatment of T2DM.

**Missing data**

For subjects unable to participate in blood drawing and examination on time, we will postpone the visit time appropriately. For subjects who insist on dropping out of the study, we will also persuade them to come for examination in advance

**References**

1. Blair, S.N., et al., *Assessment of habitual physical activity by a seven-day recall in a community survey and controlled experiments.* Am J Epidemiol, 1985. **122**(5): p. 794-804.

2. Ainsworth, B.E., et al., *Compendium of physical activities: an update of activity codes and MET intensities.* Med Sci Sports Exerc, 2000. **32**(9 Suppl): p. S498-504.

3. Chinese Diabetes Society. China guideline for type 2 diabetes (2020 edition). *Chinese Journal of Diabetes*; 2021. 13(04):316-384.

4. Xiao, H.T., et al., *Cyclocarya paliurus tea leaves enhances pancreatic β cell preservation through inhibition of apoptosis.* Sci Rep, 2017. **7**(1): p. 9155.

5. Wang, X., W. Li, and D. Kong, *Cyclocarya paliurus extract alleviates diabetic nephropathy by inhibiting oxidative stress and aldose reductase.* Ren Fail, 2016. **38**(5): p. 678-85.

6. Baig, M.A. and S.S. Panchal, *Streptozotocin-Induced Diabetes Mellitus in Neonatal Rats: An Insight into its Applications to Induce Diabetic Complications.* Curr Diabetes Rev, 2019. **16**(1): p. 26-39.
